# A smart hypochlorous acid fluorescent probe enabling Ibuprofen-release for osteoarthritis theranostics

**DOI:** 10.7150/thno.96958

**Published:** 2024-06-17

**Authors:** Zhenni Lu, Peng Wei, Hongying Peng, Libo Jiang, Peiyi Wu, Tao Yi

**Affiliations:** 1 State Key Laboratory for Modification of Chemical Fibers and Polymer Materials, College of Chemistry and Chemical Engineering, Donghua University, Shanghai 201620, China.; 2 Department of Orthopaedic Surgery, Zhongshan Hospital, Fudan University, Shanghai 200032, China.

**Keywords:** methylene blue (MB), hypochlorous acid, fluorescent probe, ibuprofen, osteoarthritis therapy

## Abstract

**Background:** Osteoarthritis (OA) standing as the most prevalent form of arthritis, closely associates with heightened levels of reactive oxygen species, particularly hypochlorous acid (HOCl). Although there are numerous probes available for detecting HOCl in the OA region, probes with dual functions of diagnostic and therapeutic capabilities are still significantly lacking. While this type of probe can reduce the time gap between diagnosis and treatment, which is clinically needed.

**Methods:** We developed a fluorescent probe (DHU-CBA1) toward HOCl with theranostics functions through the release of methylene blue (MB) and ibuprofen (IBP) in this work. DHU-CBA1 can detect HOCl with high specificity and sensitivity, releasing MB and IBP with an impressive efficiency of ≥ 95% *in vitro*.

**Results:** DHU-CBA1 exhibits good biosafety, enabling *in vivo* imaging of endogenous HOCl, along with reducing arthritis scores, improving synovitis and cartilage damage, and maintaining catabolic balance while alleviating senescence in cartilage.

**Conclusions:** This study proposes a novel approach to enhance osteoarthritis therapy by releasing IBP via a smart HOCl-enabled fluorescent probe.

## Introduction

Osteoarthritis (OA) is the most widespread arthritis, which is primarily characterized by whole articular pathological changes, such as cartilage damage, matrix loss and synovitis 1-7. With the coming of aging, it is estimated that nearly 303 million people are afflicted with OA, causing enormous social burden 8-13. The development of OA is usually connected with inflammation and oxidative stress induced by the aberrant mechanical stress in joint microenvironment 14. In the pathological process of OA, relevant research has elucidated the significant role of reactive oxygen species (ROS) in OA development 15-24. Specifically, hypochlorous acid (HOCl), generated from hydrogen peroxide (H_2_O_2_) and chloride ions (Cl^-^) catalyzed by myeloperoxidase (MPO), exhibits high reactivity. Its excessive accumulation can lead to damage to the synovial membrane 25-29. Hence, achieving in situ tracking of HOCl in the OA region and utilizing it as a biomarker to identify OA, as well as an activator for prodrugs, is of significant importance. This enables timely intervention therapy in OA, effectively curbing its progression.

To enable effective real-time tracking of HOCl in the OA region, various fluorescent probes have been developed leveraging highly sensitive fluorescence imaging technology 30-32. These fluorescent probes facilitate the tracing of HOCl in the OA area, offering a tool to elucidate the relationship between OA progression and HOCl 26, 31, 33, 34. However, current fluorescent probes are primarily limited to tracing HOCl in the OA region but cannot be utilized for treating OA 33-35. This limitation significantly impedes timely intervention in OA progression. Therefore, there is an urgent demand for the development of novel fluorescent probes with theranostic capabilities, enabling simultaneous detection of HOCl and intervention therapy for OA.

**Scheme 1:**
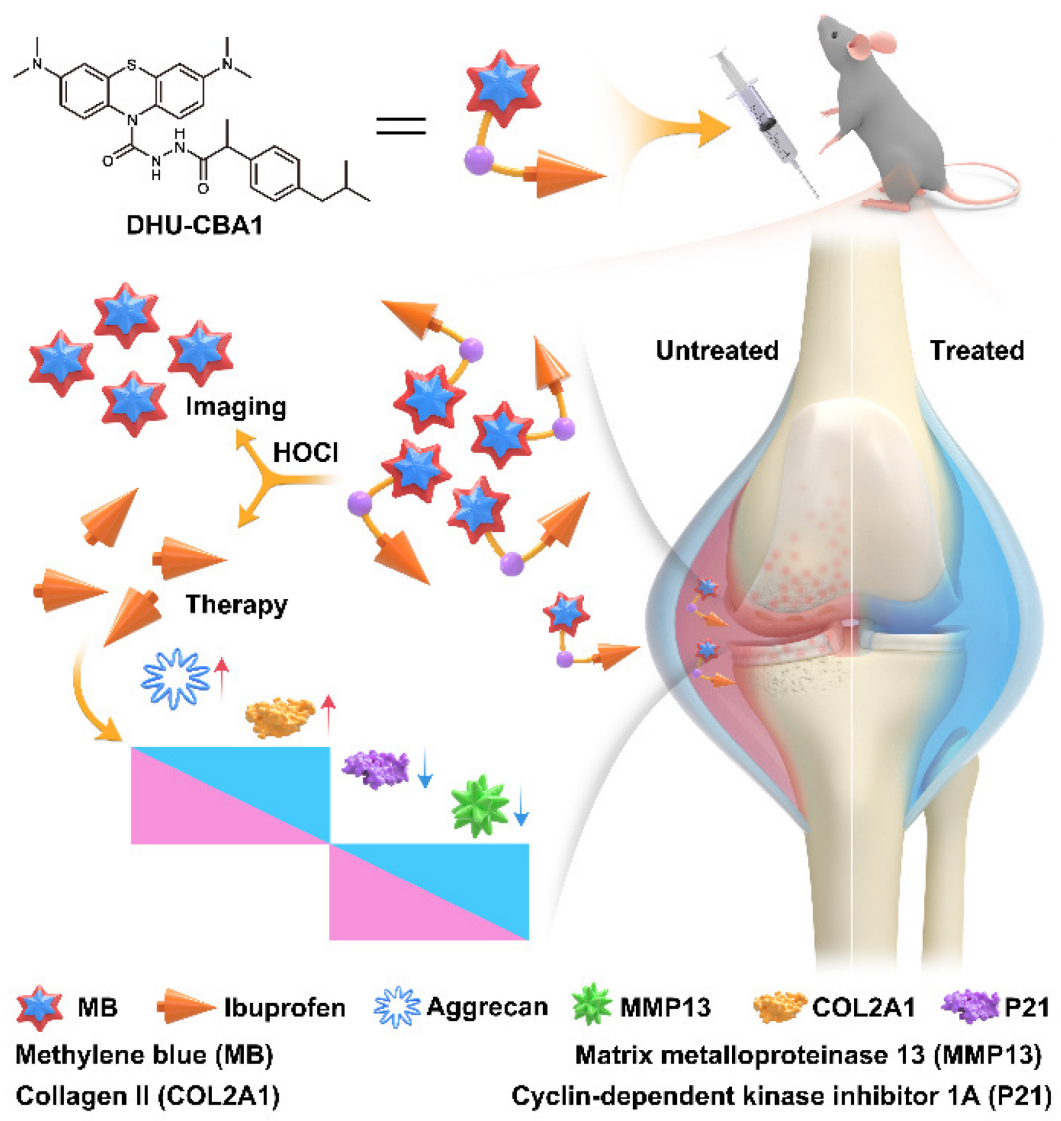
Schematic illustration of DHU-CBA1 for imaging HOCl and enhancing osteoarthritis therapy.

In our previous studies, we have successfully developed a series of HOCl-specific fluorescent probes using methylene blue (MB) structure and utilized them to investigate the presence of HOCl in diverse conditions such as the arthritis 36, tumor microenvironment 37-39, psoriasis 40, and other diseases 41-43. Herein, we designed and synthesized a fluorescent probe DHU-CBA1 by attachment of IBP (ibuprofen, a nonsteroidal anti-inflammatory drug) and MB. DHU-CBA1 could recognize HOCl rapidly with high specificity and sensitivity in less than 80 s, releasing MB and IBP along with the stimulatory generated efficiency of IBP (≥ 95%) *in vitro*. Moreover, DHU-CBA1 exhibited excellent biosafety, ensuring its utilization in living systems for OA theranostics. The *in vivo* experiments showed that DHU-CBA1 not only enabled direct fluorescence imaging of endogenously produced HOCl but also demonstrated excellent therapeutic properties during the treatment process. Therefore, the development of DHU-CBA1 provides a new option for the diagnosis and treatment of OA (Scheme 1).

## Results and Discussion

### The design and synthesis of DHU-CBA1

**Figure 1:**
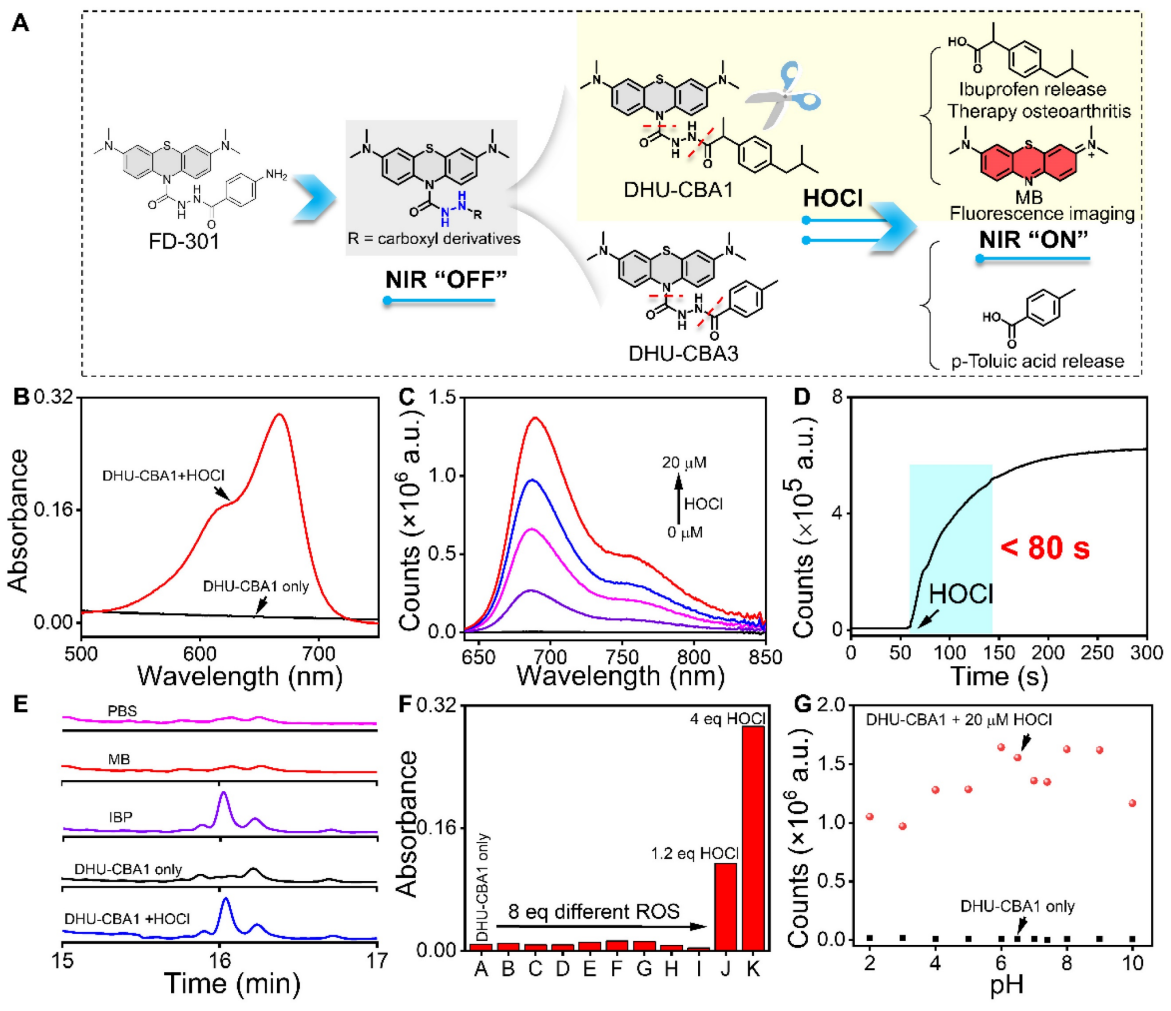
(A) The design of DHU-CBA1 and DHU-CBA3 and the proposed reaction mechanism toward HOCl. (B) The absorption spectra of DHU-CBA1 (5 μM) before and after addition of HOCl (20 μM). (C) The fluorescence spectra of DHU-CBA1 (5 μM) before and after the addition of different concentrations of HOCl (0, 5, 10, 15, 20 μM). (D) The time-dependent changes in fluorescent intensity of DHU-CBA1 (5 μM) at 686 nm after adding HOCl (20 μM). (E) HPLC analysis of the aqueous solution: PBS, 40 μM MB, 40 μM IBP, 40 μM DHU-CBA1, 40 μM DHU-CBA1+200 μM HOCl (254 nm). (F) The absorbance of DHU-CBA1 (5 μM) at 664 nm after adding different ROS (40 μM) (from A to K): DHU-CBA1 only, H_2_O_2_, TBHP, ROO^•^, NO, ^•^OH, ONOO^-^, t-BuOO^•^, O_2_^-^, 6 μM HOCl, and 20 μM HOCl. (G) The fluorescence intensity of DHU-CBA1 (5 μM) at 686 nm before and after adding 20 μM HOCl in buffer with different pH (2 - 10). Time range 0 - 300 s. λ_ex_ = 620 nm. The data was recorded in PBS (10 mM, pH = 7.4, 1‰ N, N-dimethylformamide (DMF)).

To effectively identify specific types of ROS in the OA region, the probe must exhibit excellent specificity. Additionally, to fulfill the objective of integrating diagnosis and treatment, the probe should be capable of releasing specific therapeutic drugs in response to the specific ROS. OA patients often resort to using nonsteroidal anti-inflammatory drugs (NSAIDs) to alleviate symptoms due to their analgesic and anti-inflammatory effects. Among the various NSAIDs available, those containing phenylacetic acid structures, exemplified by IBP, possess a single active site (typically a carboxyl group) and demonstrate favorable therapeutic effects for OA. This makes them suitable prototype drugs for the development of probes. In our previous work, we successfully developed an MPO fluorescent probe (FD-301) through the selective recognition of HOCl. Notably, FD-301 possesses the capacity to release carboxyl compounds upon activation (Figure 1A). Utilizing this characteristic, we designed and synthesized DHU-CBA1 and DHU-CBA3 (comparative compound) through structural modification. The detailed synthetic procedures and structural characterization data are provided in the Supplementary Information (Scheme S1).

### The characterization of the probes toward HOCl

The response properties of the probes DHU-CBA1 and DHU-CBA3 toward HOCl *in vitro* were initially examined by means of absorption and fluorescence spectroscopy in the PBS solutions (10 mM PBS, pH = 7.4, 1‰ N, N-dimethylformamide (DMF)). The absorption of DHU-CBA1 increased predictably with 0 - 20 μM HOCl with the maximum absorbance at 664 nm (Figure S2A and Figure 1B). Similarly, for DHU-CBA3, signals exhibited a characteristic absorption peak at 664 nm upon treatment with 10 μM HOCl (Figure S1A). Furthermore, the fluorescence intensity of 5 μM DHU-CBA1 or 5 μM DHU-CBA3 gradually increased at 686 nm with rising HOCl concentrations (Figure 1C and Figure S1B). Subsequently, we investigated the response time of DHU-CBA1 and DHU-CBA3 toward HOCl. As illustrated in Figure 1D, the fluorescence of DHU-CBA1 at 686 nm immediately increased in less than 80 s. Meanwhile, DHU-CBA3 could reach equilibrium within 150 s (Figure S1C). The products resulting from the reactions of DHU-CBA1 or DHU-CBA3 with HOCl were analyzed using high-performance liquid chromatography (HPLC) and high-resolution mass spectrometry (HRMS). In the HPLC tests, two new peaks were observed for DHU-CBA1 at 11.26 and 16.02 min, corresponding to the free drug of MB and IBP fragment, respectively (Figure 1E and Figure S3A). For DHU-CBA3, HPLC confirmed the production of MB and p-Toluic acid (pTA) at 11.26 and 11.79 min, respectively (Figure S6A). HRMS further confirmed the production of these fragments (for DHU-CBA1: [MB - Cl^-^]^+^ calculated for C_16_H_18_N_3_S^+^: 284.1216, found: 284.1225 and [IBP - H]^-^ calculated for C_13_H_17_O_2_^-^: 205.1234, found: 205.1227; for DHU-CBA3: [MB - Cl^-^]^+^ calculated for C_16_H_18_N_3_S^+^: 284.1216, found: 284.1231 and [pTA - H]^-^ calculated for C_8_H_7_O_2_^-^: 135.0451, found: 135.0440) (Figures S4, S5, S7 and S8). Meanwhile, the release calculated efficiency of IBP and pTA was 97% and 78% respectively, indicating the remarkable release ability (Figures S3B, S3C, S6B, and S6C). Following the similar way, the release efficiency of MB (95%, Figure S3D) illustrated that the relationship of IBP and MB was matched. Comparing with DHU-CBA1 and DHU-CBA3, we found that the substantial steric hindrance around the recognition site of compound DHU-CBA3 results in a slower reaction rate and lower release efficiency compared to compound DHU-CBA1. Additionally, detection limit tests of compound DHU-CBA1 revealed that it possesses higher sensitivity (Figure S2B and S2C, 3.72 nM based on the 3σ/k method). The above results proved that DHU-CBA1 could respond with HOCl in solution, releasing MB and related compounds with carboxyl functional group with high efficiency.

A series of ROS containing H_2_O_2_, TBHP, ROO^•^, NO, ^•^OH, ONOO^-^, t-BuOO^•^, O_2_^-^ were applied to explore the selectivity of DHU-CBA1 and DHU-CBA3 in PBS. Notably, only HOCl could activate the two probes significantly, with the enhancement of the absorption (at 664 nm) and fluorescence intensity (686 nm) > 30-fold and > 100-fold, respectively. Moreover, a significantly lower concentration of 6 μM and 3 μM HOCl could activate the probes (5 μM), while all other ROS showed minimal variation, demonstrating the high specificity and sensitivity of the probes toward HOCl (Figure 1F, Figures S1D, S9A-S9C, and S10A-S10C). We then explored the influence of omnipresent various anions (CH_3_COO^-^, CO_3_^2-^, SO_4_^2-^, F^-^, Cl^-^, I^-^, NO_2_^-^, S_2_O_3_^2-^), cations (NH_4_^+^, Na^+^, Mg^2+^, Al^3+^, K^+^, Ca^2+^, Fe^3+^, Cu^2+^, Ni^2+^), and amino acids (Leu, Pro, Gly, Gln, Glu, Met, Lys, Trp, Ser, Thr, Asp, Ile, Val, His, Ala, Cys, Phe, Asn, Tyr, Arg) (all of these analysts were 150 μM) toward DHU-CBA1 (5 μM). The results (Figures S11A-S11C and S12A-S12C) indicated that all these analysts could not react with DHU-CBA1. Meanwhile, DHU-CBA1 could maintain stable and react with HOCl in the pH range of 2 to 10 (Figure 1G and Figure S13A-S13C), revealing that the probe DHU-CBA1 could be used for the biological detection of HOCl with high selectivity and sensitivity. The above results proved that the probe could respond with HOCl in solution, releasing MB for imaging and IBP for treatment, laying a foundation for combining the detection of HOCl level in OA and the treatment of OA.

### The *in vitro* biosafety of probes

**Figure 2:**
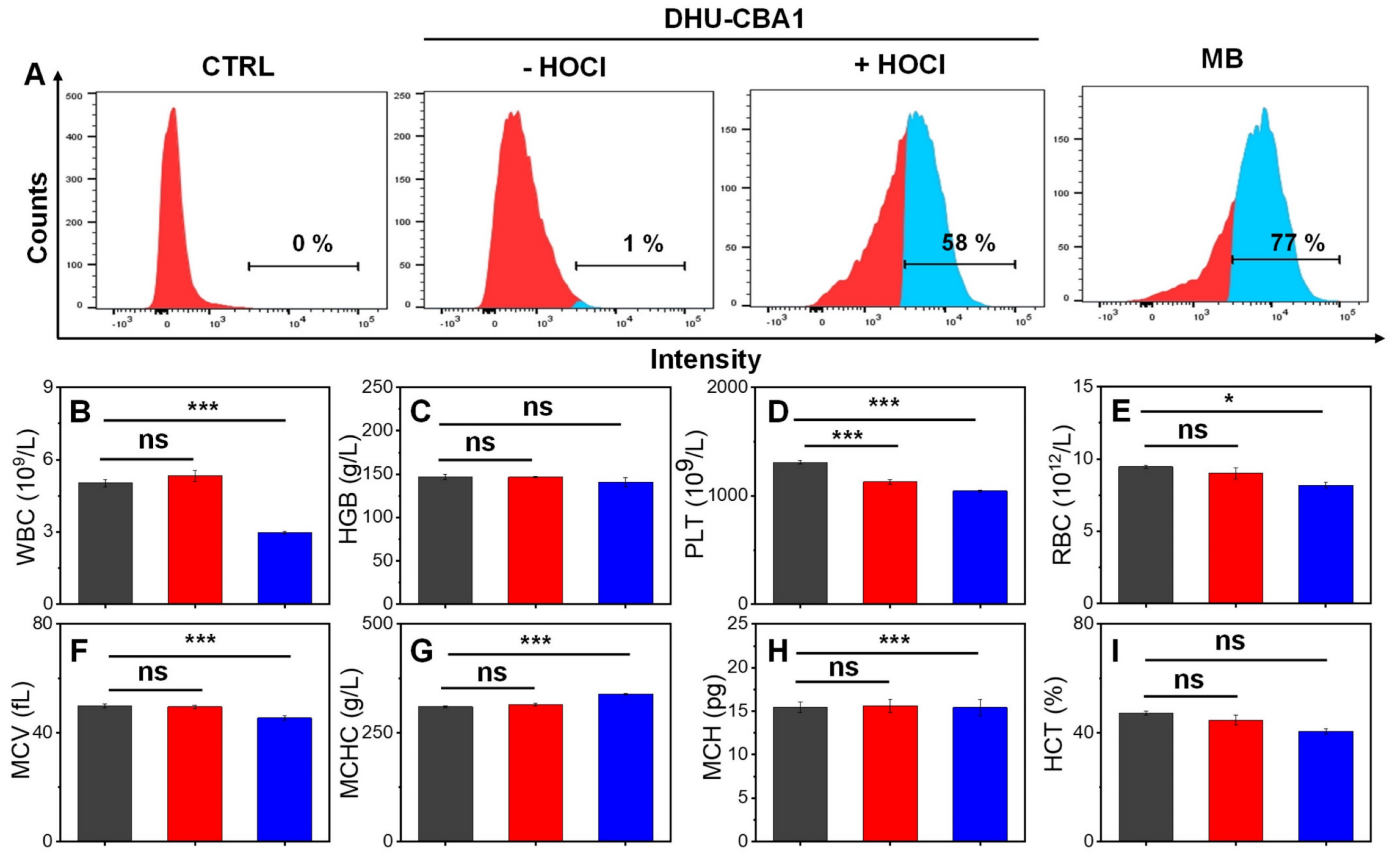
(A) Flow cytometry analysis of chondrocytes cells uptake and activation by medium (CTRL), 10 μM DHU-CBA1, 10 μM DHU-CBA1+ HOCl, and 10 μM MB, respectively. Toxicity assessments of healthy mice received intraperitoneal injections of (black column) PBS, (red column) DHU-CBA1(0.5 mM, 100 μL), and (blue column) DHU-CBA3 (0.5 mM, 100 μL) treatment every three days for one month. (B-I) Blood routine examination, including white blood cell (WBC), hemoglobin (HGB), platelet (PLT), red blood cell (RBC), mean corpuscular volume (MCV), mean corpuscular hemoglobin concentration (MCHC), mean corpuscular hemoglobin (MCH), hematocrit (HCT). Each group performed three independent parallel experiments (n = 3). Values are the mean ± SD. *p < 0.05, **p < 0.01, ***p < 0.001.

At the initiation of the biological experiments, we initially assessed the cellular uptake behavior of the probes (Figure 2A). Chondrocytes were initially incubated with DHU-CBA1 or DHU-CBA3, followed by the external addition of HOCl. The cellular uptake behavior was evaluated by monitoring the production of MB after response. As shown in Figure 2A and Figure S14A-S14C, the content of MB-positive chondrocytes of DHU-CBA1 or DHU-CBA3 increased to 58% and 60%, respectively, much higher than the control group. Additionally, the observed content was very similar to the group where MB was independently incubated (77%). These data confirmed that DHU-CBA1 and DHU-CBA3 could be uptaken by chondrocytes and respond toward HOCl. Meanwhile, we investigated the MB release properties of DHU-CBA1 using a confocal laser scanning microscope (CLSM). The CLSM images, as shown in Figure S16, revealed no significant fluorescence signal in the control groups treated with DHU-CBA1 (10 μM) for 1 h compared to those treated with F12 Ham's medium. However, further incubation with exogenous HOCl (40 μM) for 15 min resulted in a remarkable increase in fluorescence intensity. This indicates that DHU-CBA1 can be effectively used to identify HOCl at the cellular level through the release of MB. As a crucial criterion, assessing the *in vivo* biocompatibility of probes holds significant importance for their subsequent clinical applications. We thus examined the effect of DHU-CBA1, DHU-CBA3 and pTA on chondrocytes viability. After co-incubation with various concentrations (5∼40 μM) of probes for 24 h, the CCK-8 assay showed that chondrocytes of all groups gave favorable cells viability (more than 80%) (Figure S15A-S15C). Additionally, the dead/live assay further showed no cytotoxicity of DHU-CBA1 at different concentration (10, 20, 40 μM) and DHU-CBA3 (40 μM) for 12 h and 24 h (Figure S15D). In terms of routine blood analysis, treatment with probes DHU-CBA1 (0.5 mM, 100 μL) and DHU-CBA3 (0.5 mM, 100 μL) had no discernible impact toward WBC, HGB, PLT, RBC, MCV, MCHC, HCT, and MCH (Figure 2B-2I), indicating good hemocompatibility. Therefore, DHU-CBA1 and DHU-CBA3 had good biosafety *in vitro*, which deserved the further application in osteoarthritis therapy.

### The HOCl-responsive behavior of probes in the OA model

**Figure 3:**
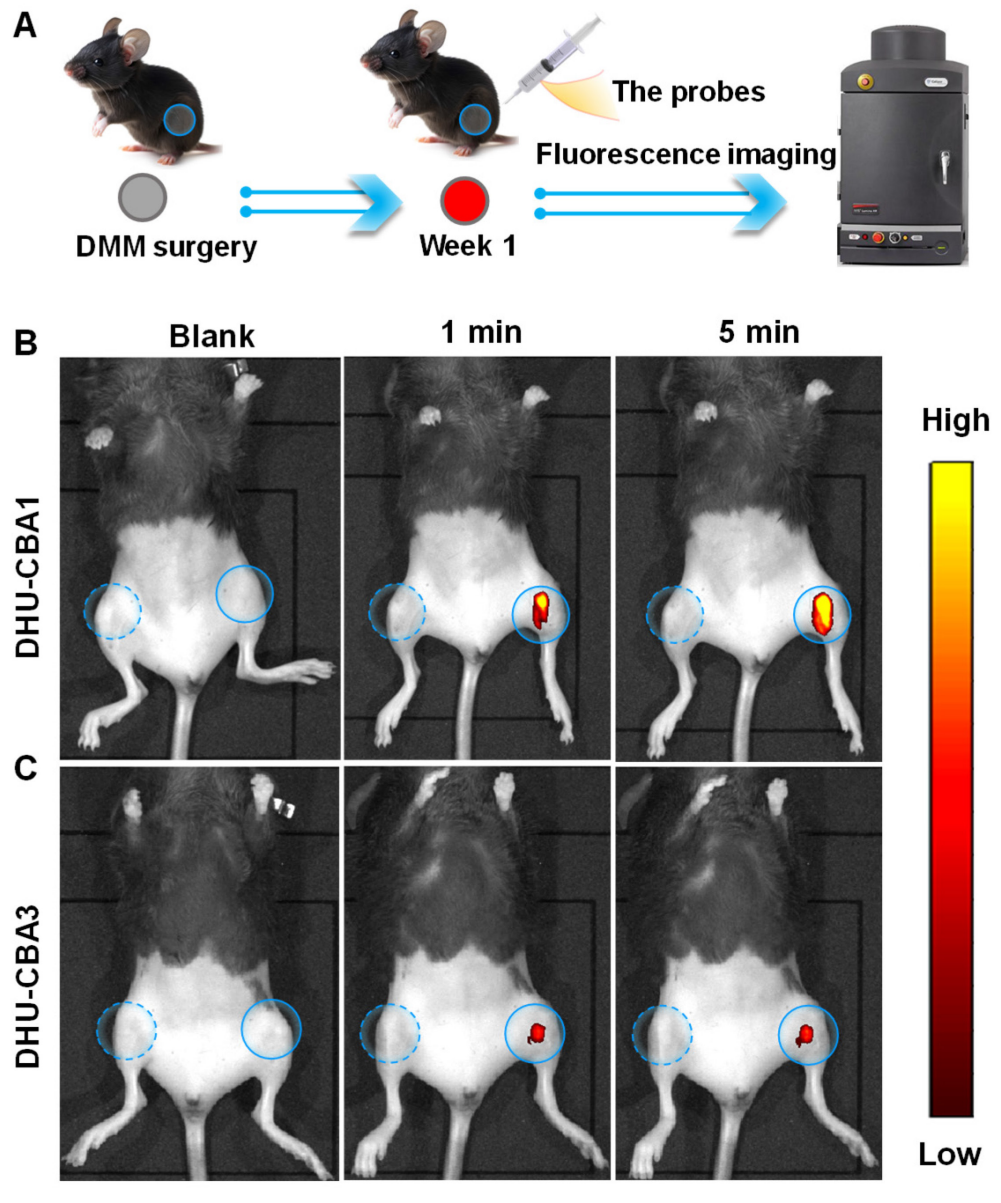
(A) Schematic illustration of probes for the fluorescence images with (B) DHU-CBA1 and (C) DHU-CBA3 (10 μL × 0.5 mM) for different times (0, 1, 5 min) of OA model *in vivo*.

Excessive ROS, particularly HOCl, have been identified as critical features in inflammatory joint diseases 26, 31, 34. Surgical joint destabilization in mice, a method used to establish an OA model, has been shown to lead to the release of excessive HOCl 26, 34. Therefore, we performed surgical destabilization of the medial meniscus (DMM) to create a mice OA model for further studying the response behavior of DHU-CBA1 or DHU-CBA3 toward HOCl *in vivo* (Figure 3A). The fluorescence of MB was utilized to identify the response and drug activation, enabling us to track the probes' response in the knee articular cavity using an *in vivo* bioluminescence imaging (IVIS). Seven days after DMM surgery, the intra-articular injection of DHU-CBA1or DHU-CBA3 (0.5 mM, 10 μL) was performed for the bilateral knee articular cavities (Figure 3B and Figure 3C).

Following injection, only the surgically altered left knee exhibited notable fluorescence signals within 1 minute, while no fluorescence signal was observed in the normal right knee. The fluorescence signal of probes reached maximum brightness at 5 min and remained stable for 20 min (Figure S17A-D). The released substrate is rapidly metabolised by the body (MB: t1/2 < 2 h 44, 45, IBP: t1/2 < 24 h 46). However, in *in vivo* imaging, the degree of fluorescence intensity changes between DHU-CBA1 and DHU-CBA3 is different. This is attributed to the differences in the reaction of DHU-CBA1 and DHU-CBA3 toward HOCl. As shown in Figure 1A, DHU-CBA3 exhibits greater steric hindrance, which hinders its reaction with HOCl. This was validated *in vitro* experiments as depicted in Figure 1 and S1, where after responding to the same concentration of HOCl, the absorption and fluorescence changes of DHU-CBA1 were more significant than DHU-CBA3. These findings illustrated that DHU-CBA1 and DHU-CBA3 exhibited exceptional sensitivity in detecting endogenously produced HOCl within OA areas at the *in vivo* level.

### DHU-CBA1 intervention in OA model development

**Figure 4:**
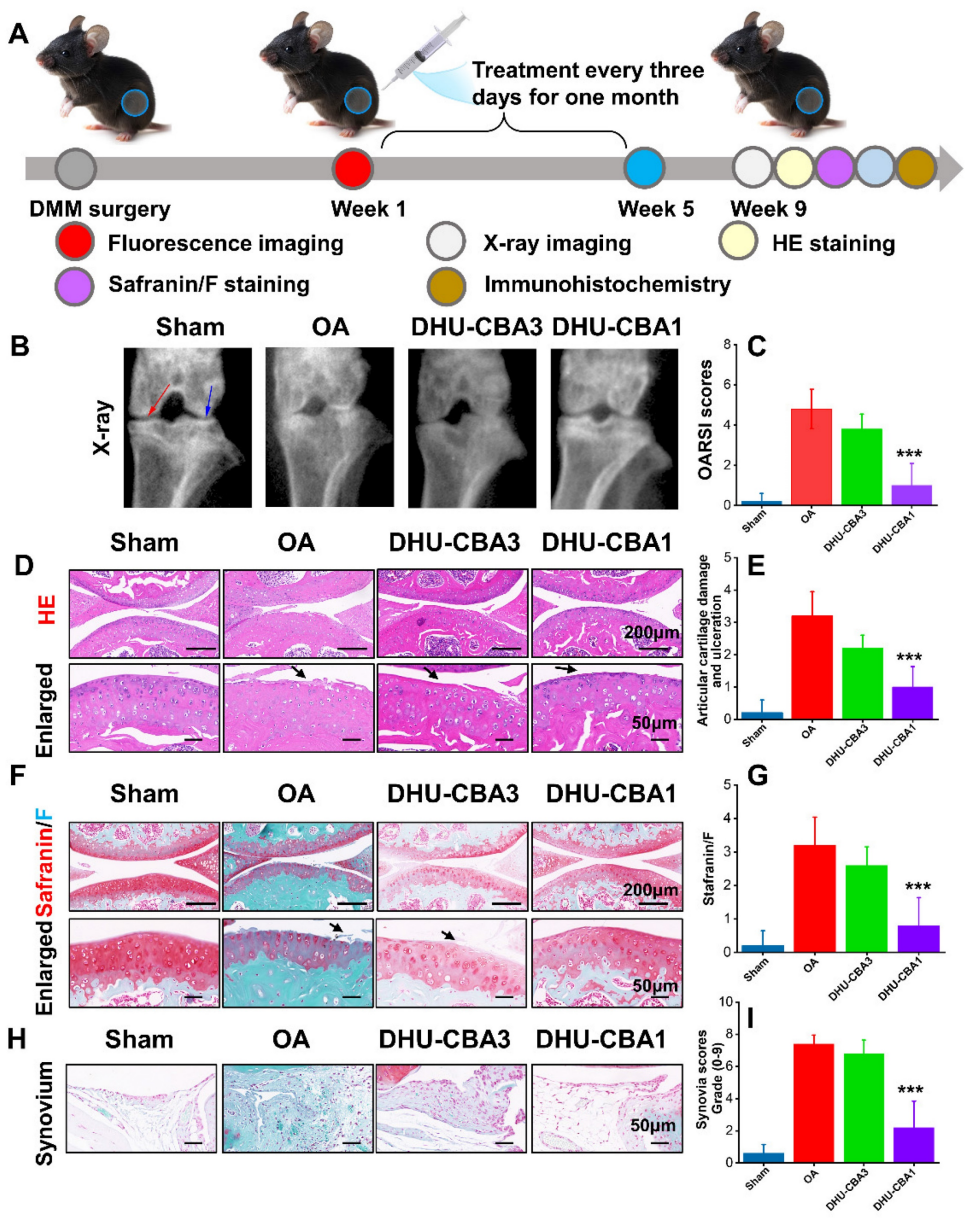
(A) Schematic illustration of the treatment in the OA model. The drug treatment evaluation of OA: the X-ray imaging (B), Osteoarthritis Research Society International (OARSI) scores (C), hematoxylin and eosin (HE) staining (D) and articular cartilage damage and ulceration (E), Safranin/F staining (F) and related scores (G), Synovium (H) and related scores (I). n = 5 mice per group. Data are presented as means ± SD. *p < 0.05, **p < 0.01, ***p < 0.001.

Encouraged by the *in vivo* response of the probes toward HOCl, we designed an experiment to assess the therapeutic impact of the probes on OA treatment (Figure 4A). Two months post-DMM surgery, we initially assessed treatment efficacy on OA progression through X-ray analysis. As depicted in Figure 4B, compared to the sham group, the OA group exhibited narrowed joint space (the coloured red and blue arrows) and severe articular surface degeneration. Following intra-articular injection with probes, which is the most widely used for OA, DHU-CBA3 marginally ameliorated OA, while DHU-CBA1 demonstrated significant improvement. These were because DHU-CBA1 released drug IBP after responding with HOCl, while DHU-CBA3 consumed HOCl itself but no anti-inflammatory drug was released. Subsequent HE and Safranin/F assays further indicated that DHU-CBA1 mitigated superficial cartilage destruction, proteoglycan loss, and narrowed space (Figure 4D and 4F). Moreover, Osteoarthritis Research Society International (OARSI) scores, articular cartilage damage and ulceration scores, and Safranin/F staining scores were notably lower in the DHU-CBA1 group compared to the OA and DHU-CBA3 groups (Figure 4C, 4E, and 4G). Synovitis is a critical indicator of OA development. As observed in Figure 4H, the DHU-CBA1 group exhibited fewer synovial lining layers and lower density of resident cells in the synovium, comparable to the OA and DHU-CBA3 groups. The total synovitis scores also indicated significant synovitis improvement in the DHU-CBA1 groups (Figure 4I). However, DHU-CBA3 only demonstrated slight improvement in cartilage repair and synovitis evaluation, indicating limited treatment efficacy by released MB only. These results underscore DHU-CBA1's robust efficacy in OA treatment via intra-articular injection, stemming from the released IBP.

### DHU-CBA1 preserves catabolic balance and mitigates cartilage senescence

The subsequent investigation aimed to elucidate the mechanism underlying the rescue of OA development through DHU-CBA1 treatment. Cartilage senescence and extracellular matrix (ECM) dynamics are pivotal factors in OA progression 47-49, and were therefore assessed via immunohistochemical staining in this study.

**Figure 5:**
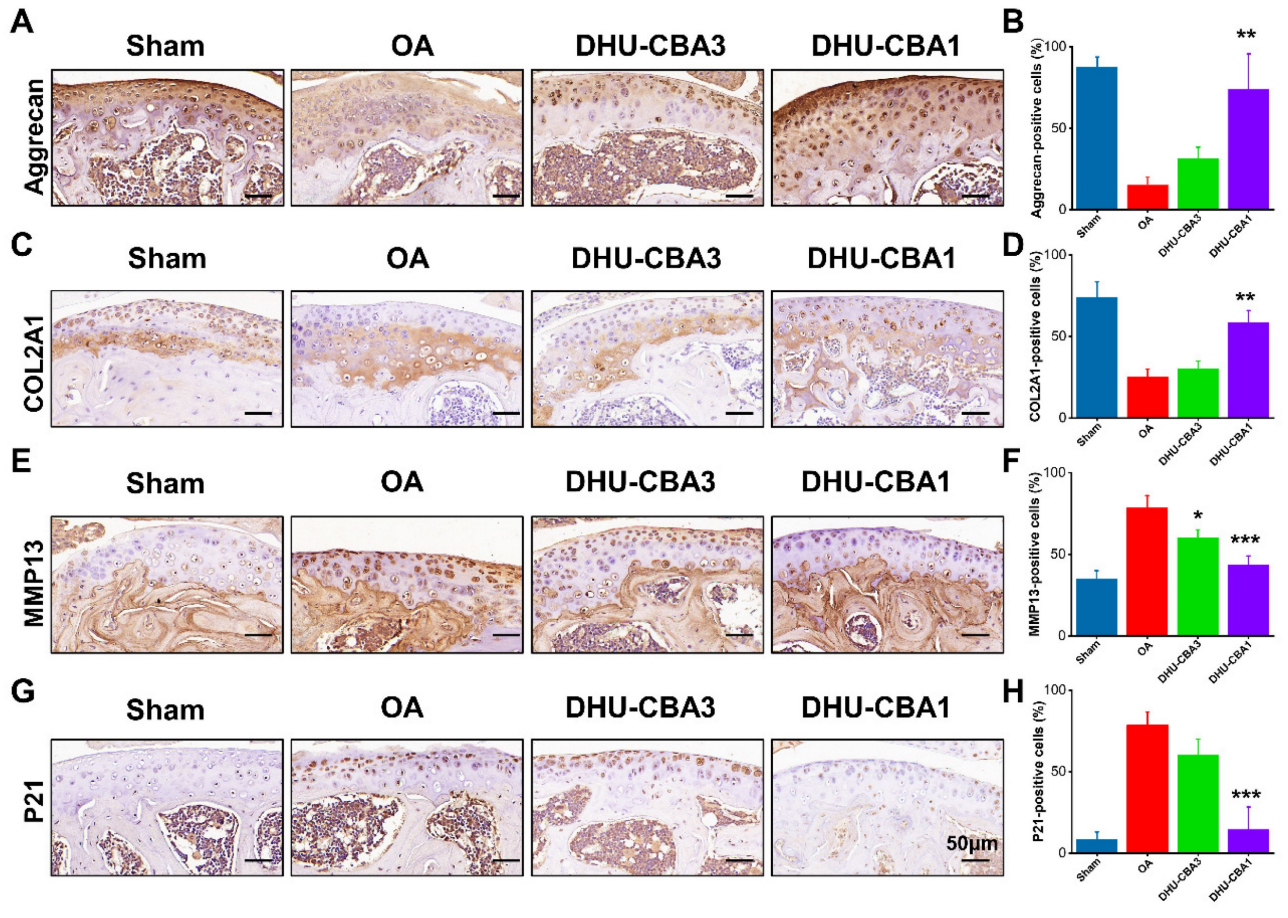
(A) Representative immunohistochemistry staining images of ECM markers (Aggrecan) and (B) statistical analysis of Aggrecan-positive cells. (C) Representative immunohistochemistry staining images of ECM markers (COL2A1) and (D) statistical analysis of COL2A1-positive cells. (E) Representative immunohistochemistry staining images of matrix-degrading enzyme (MMP13) and (F) statistical analysis of MMP13-positive cells. (G) Representative immunohistochemistry staining images of senescence marker (P21) and (H) statistical analysis of P21-positive cells. From left to right: Sharm, OA, DHU-CBA3, and DHU-CBA1 groups. n = 3 mice per group. Data are presented as means ± SD. *p < 0.05, **p < 0.01, ***p < 0.001.

Remarkably, there was a significant increase in cells positive for ECM markers (aggrecan and COL2A1) in the DHU-CBA1 group compared to the OA and DHU-CBA3 groups (Figure 5A-5D). Correspondingly, the expression of the ECM degradation marker MMP13 and representative senescence marker P21 was markedly elevated following DMM surgery compared to the sham group. However, these indicators were slightly downregulated with DHU-CBA3 treatment and predominantly rescued with DHU-CBA1 treatment (Figure 5E-5H). Collectively, these findings demonstrate that DHU-CBA1 preserves catabolic balance and mitigates cartilage senescence, thereby attenuating OA progression.

## Conclusion

In summary, we have successfully developed a smart HOCl-responsive probe, DHU-CBA1, consisting of MB and IBP, aimed at imaging HOCl companied with enhancing OA treatment. DHU-CBA1 exhibited rapid activation by HOCl within a remarkably short time of 80 s, demonstrating favorable selectivity and sensitivity. DHU-CBA1 achieved a rapid IBP release ratio of ≥ 95% upon exposure to HOCl *in vitro*. Moreover, DHU-CBA1 displayed excellent biosafety, which is a crucial indicator for subsequent highly efficient and precise OA treatment. Furthermore, DHU-CBA1 exhibited superiority in reducing arthritis scores, improving synovitis and cartilage damage, preserving catabolic balance, and mitigating cartilage senescence.

## Supplementary Material

Supplementary methods and figures.

## Abbreviations

OA: osteoarthritis
HOCl: hypochlorous acid
PBS: phosphate-buffered saline
s: second
min: minute
h: hour
MB: methylene blue
WBC: white blood cell
PLT: platelet
RBC: red blood cell
MCV: mean corpuscular volume
MCHC: mean corpuscular hemoglobin concentration
HCT: hematocrit
MCH: mean corpuscular hemoglobin
HGB: hemoglobin
