# Supplementary material for: A smart hypochlorous acid fluorescent probe enabling Ibuprofen-release for osteoarthritis theranostics
Source: Theranostics. 2024 Jun 17;14(10):3900–8. doi: 10.7150/thno.96958 (PMC11234266; doi:10.7150/thno.96958)
Supplement: Supplementary file 1 — Supplementary methods and figures. [file thnov14p3900s1.pdf]

Supporting Information

A smart hypochlorous acid fluorescent probe enabling Ibuprofen-release for osteoarthritis theranostics

Zhenni Lu[1], Peng Wei[1], Hongying Peng[1], Libo Jiang[2*], Peiyi Wu[1*], Tao Yi[1*]

[1]State Key Laboratory for Modification of Chemical Fibers and Polymer Materials College of Chemistry and Chemical Engineering, Donghua University, Shanghai 201620, China

[2]Department of Orthopaedic Surgery, Zhongshan Hospital, Fudan University, Shanghai 200032, China

*To whom correspondence should be addressed: Tao Yi.

E-mail address(es): jiang.libo@zs-hospital.sh.cn, wupeiyi@dhu.edu.cn, yitao@dhu.edu.cn

Content

1. Materials and methods 2

2. Supporting figures 7

3. References 24

1. Experiment Section

1.1 Materials

Unless specific requirements, all the organic solvents are purchased and used without additional purifications. Cell counting kit-8 (CCK-8) was purchased from MedChemExpress, USA. Safranin/F fast green staining was obtained from Solarbio, Beijing, China. Hematoxylin and eosin (HE) staining in accordance with the manufacturer's protocol. Aggrecan (13880-1-AP, proteintech), MMP13 (18165-1-AP, proteintech), collagen II (COL2A1, 28459-1-AP, proteintech), P21 (10355-1-AP, proteintech) were purchased from Wuhan, China. Methylene blue (MB), Ibuprofen (IBP), p-Toluic acid (pTA), Oxalyl chloride, and other common chemicals were acquired and used directly without further purification.

1.2 Instruments

^{1}H NMR (400 MHz) and ^{13}C NMR (100 MHz) spectra were recorded with a Bruker AV400 nuclear magnetic resonance spectrometer with DMSO-d_6 as the solvent. Proton chemical shifts are reported in parts per million downfield from tetramethylsilane (TMS), with tetramethylsilane (δ = 0.0 ppm) or DMSO-d_6 (39.52 ppm for ^{13}C) as the chemical shift standard. High-resolution mass spectra (HRMS) were observed on Bruker Micro TOF II instrument using electrospray ionization (ESI) or Bruker Compact QTOF-HRMS. High-performance liquid chromatography (HPLC) was acquired utilizing an Agilent 1200 Series system. UV-vis absorption spectra were obtained using a Shimadzu UV-2600 spectrophotometer at a medium scanning rate and quartz cuvettes with a path length of 1 cm. Fluorescence spectra were recorded at room temperature using an Edinburgh FLS 1000 spectrometer with an Xe lamp as the excitation source. The lived / dead cytotoxicity assay images were taken by a confocal laser scanning microscope (CLSM, FV3000, Olympus, JPN). The fluorescence imaging were carried out on a commercial IVIS Lumina Ⅲ small animal in vivo fluorescence imaging system (PerkinElmer, USA).

1.3 Material synthesis and preparation

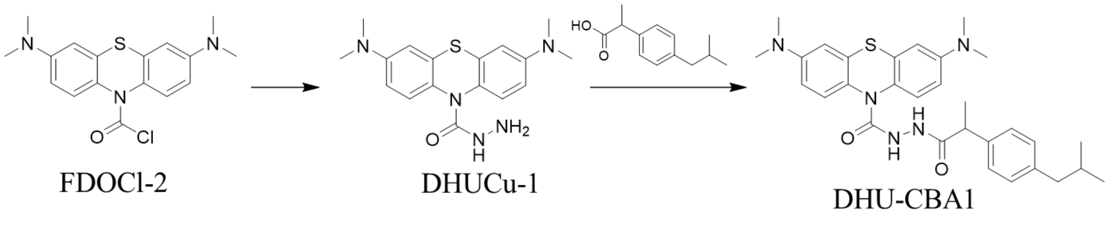

Scheme S1. The general synthetic route of probe DHU-CBA1.

DHU-CBA3, FDOCl-2, and DHUCu-1 were synthesized according to our reported procedure [1-3]. The general synthetic procedure of the probe (DHU-CBA1) is shown in Scheme S1. Ibuprofen (0.60 g, 2.9 mmol, 1 eq) was dissolved in 10 mL CH_2Cl_2 and stirred at 0 ℃, oxalyl chloride (0.74 mL, 8.7 mmol, 3

eq) was added dropwise, and the mixture was stirred in an ice-water bath and under N_2 atmosphere for 0.5 h. The solvent was removed under reduced pressure. The obtained residue was dissolved in CH_2Cl_2 and added dropwise to a mixture of DHUCu-1 (0.50 g, 1.46 mmol, 1 eq), 10 mL CH_2Cl_2, and sodium ccarbonate (0.28 g, 2.6 mmol, 1.5 eq). The mixture was stirred in an ice-water bath for 0.5 h and then stirred at room temperature until the reaction was complete. It is extracted with ethyl acetate and then purified by silica gel column chromatography using eluents of petroleum ether/ethyl acetate (1: 2). Then the compound DHU-CBA1 as white solids in yields of 75% (0.578 g). ^{1}H NMR (400 MHz, DMSO-d_6) δ 9.80 (s, 1H), 8.02 (s, 1H), 7.35 (d, J = 8.8 Hz, 2H), 7.23 (d, J = 7.6 Hz, 2H), 7.06 (d, J = 7.2 Hz, 2H), 6.71 - 6.65 (m, 4H), 3.60 (dd, J = 14.0 Hz, 6.8 Hz, 1H), 2.88 (s, 12H), 2.39 (d, J = 6.8 Hz, 2H), 1.82 - 1.75 (m, 1H), 1.33 (d, J = 6.8 Hz, 3H), 0.84 (d, J = 6.4 Hz, 6H). ^{13}C NMR (100 MHz, DMSO-d_6) δ 172.8, 154.8, 148.6, 139.2, 138.7, 132.9, 128.6, 127.9, 127.1, 127.0, 111.2, 110.2, 44.2, 42.4, 40.2, 29.6, 22.2, 18.4. HRMS (ESI): $[M+H]^+$ calcd for $C_{30}H_{38}N_5O_2S^+$: 532.2741; found: 532.2735 (Figure S18-S21).

1.4 Preparation of probes and analytes

Reserve solutions of probes (5 mM) were prepared in dimethyl formamide (DMF), and then diluting with PBS buffer (10 mM, pH 7.4) to the test concentration. All the characterizations are managed at room temperature. Other analytes were made in ddH_2O on the basis of our reported method and specifics were as follows [1, 4, 5]. HOCl was obtained from sodium hypochlorite solution (0.1 M, H_2O-medium). H_2O_2 was taken by a 30% H_2O_2 solution. $^\bullet$OH (Hydroxyl radical) was carried out on Fenton reaction (H_2O_2: $FeCl_2$ = 1: 10) and the concentration of $^\bullet$OH was equivalent to the concentration of H_2O_2. TBHP (tert-butyl hydroperoxide) was produced from 70% TBHP solution in ddH_2O. ROO$^\bullet$ was acquired from dissolving 2, 2'-azobis (2-amidinopropane) dihydrochloride in ddH_2O. NO was conducted with dissolving SNP (sodium nitroferricyanide (III) dihydrate) in ddH_2O. O_2^- was produced by dissolving KO_2 (potassium superoxide) in DMSO. $ONOO^-$ was obtained from 3-morpholinosydnonimine hydrochloride. t-BuOO$^\bullet$ was taken by adding TBHP within 10 equiv. of ferrous chloride and the concentration of t-BuOO$^\bullet$ was equal to the TBHP concentration.

1.5 Procedure of selectivity studies in vitro

The probes DHU-CBA1 and DHU-CBA3 were treated with various concentrations of ROS in PBS (1‰ DMF) for an adequate 30 min along with the published methods. Unless otherwise noted, for all fluorescent measurements, the excitation wavelength was 620 nm and the emission wavelength was 686 nm and collected from 630 nm to 850 nm. The relevant parameters were set as the same as the fluorescence response experiment.

1.6 Detection limit

The detection limit (3σ/k) was calculated based on the linear relationship between the fluorescence intensity at 686 nm and the concentration of HOCl. σ is the standard deviation of the blank measurement (n = 15) and k is the slope of the fluorescence intensity versus HOCl concentration.

1.7 Drug release of HPLC analysis

HPLC was acquired from an Agilent 1200 Series system utilizing a C18 column at 30 ℃. The eluant was a mixture of HPLC-grade CH_3CN and H_2O (1‰ trifluoroacetic acid). The relevant parameters were set as follows: flow rate 1 mL/min. Solvent A (CH_3CN) and solvent B (H_2O) were washed within 30 min according to different gradients. Absorbance wavelengths were 210 nm, 254 nm, 290 nm, and 664 nm, successively. HPLC experiments were conducted after the MB for 11.26 min, IBP for 16.02 min, and p-pTA for 11.79 min.

1.8 Primary articular chondrocytes culture

Primary articular chondrocytes were extracted from the articular surface of knee joint of 1-week-old Sprague Dawley (SD) rats. Articular cartilage was shredded and then digested in 0.2% collagenase II (Sigma-Aldrich) at cell incubator (5% CO_2, 37 °C) for 4 h. After filtration and centrifugation, the chondrocytes were cultured with F12 Ham medium containing 10% fetal bovine serum (FBS) as well as 1% of antibiotics, followed by incubation in the incubator.

1.9 Cell viability assay

For the drug toxicity, the chondrocytes were seeded into a 96-well plate overnight, followed by incubation with pTA, DHU-CBA3, and DHU-CBA1 at different concentrations (0 ~ 40 μM) for 24 h. Then, the medium containing 10% Cell Counting Kit-8 (CCK-8, MedChemExpress, USA) was added and incubated without light for 1h. Afterward, the cell viability was determined at 450 nm absorbance (A450), and calculated as follows:

Cell viability (%) = $(A - A_0) / (A_s - A_0) \times 100$ %.

The A, A_0, and A_s respectively represented the A450 value of only medium group, the experimental group, and control group.

1.10 Cell live/dead assay

After being cultured with DHU-CBA3 and DHU-CBA1 for 12 h and 24 h, the cells were stained with calcein-AM and PI solutions for 30 min at 37 °C using a live/dead cytotoxicity assay kit (Invitrogen). After washing three times (5 min each time) with PBS, the samples were observed under a laser confocal microscope.

1.11 Probes response *in vitro*

The MB release properties of DHU-CBA1 and DHU-CBA3 in vitro were studied in chondrocytes cells. Cells (5×10^8 per mL) were separately plated on 20 mm glass bottom cell culture dish and allowed to adhere for 12 h. The stock solution of DHU-CBA1 in DMF (10 mM) was diluted with basic F12 Ham medium with final concentration of 10 μM. The cells were then incubated with different analytes for a pre-set time at 37 °C. After incubation 1 h, the cells were washed one time with PBS. CLSM imaging was performed on Leica SP 8 Confocal Laser Scanning Microscope with a 63 × oil-immersion objective lens for cells. λ_{ex} = 633 nm; red channel: 700 ± 50 nm.

1.12 Blood routine assay

PBS, DHU-CBA1, or DHU-CBA3 (0.5 mM, 100 μL) were injected into the intraperitoneal of healthy mice (n = 3). After every three days for one month of injection, ocular vein blood was taken for a blood routine.

1.13 Animal model construction

Guiding principles used for animal treatment and care closely followed the recommendations of the animal ethics committee of Zhongshan Hospital at Fu Dan University (Shanghai, China, No: 2024-036). All C57BL/6 mice (aged eight weeks old, female) were purchased from JSJ company (Shanghai, China). Osteoarthritis (OA) mouse models were established by surgical destabilization of medial meniscus (DMM). Briefly, after anesthesia with pentobarbital, left knee joint capsules were incised medial to the patellar tendon, and then transecting medial meniscotibial ligaments using microsurgical scissors. The joint capsule and skin were accordingly closed. The mice with sham surgery were conducted with arthrotomy, followed by suture without meniscotibial ligament transection.

1.14 Probes response in OA model

The drug release behavior of the probes DHU-CBA1 and DHU-CBA3 was detected in OA mice. The left knee joint was conducted with DMM surgery, and the right knee joint was conducted with sham surgery. After one week, the left and right knee joint were injected nearly 10 μL of DHU-CBA1 (0.5 mM) and DHU-CBA3 (0.5 mM), respectively. Subsequently, the fluorescence of every mouse was collected by in vivo bioluminescence imaging.

1.15 Osteoarthritis treatment

All mice were randomly divided into four groups (n = 5) as follows: sham+PBS (sham), DMM+PBS (OA), DMM+DHU-CBA3 (DHU-CBA3), DMM+DHU-CBA1 (DHU-CBA1) groups. Sham group and OA group mice were treated by intra-articular administration of OA solution (0.5%), while treatment group mice were treated with DHU-CBA3 (0.5 mM) and DHU-CBA1 (0.5 mM) every three days for one month. After two months, the knee joints were harvested and photographed via X-ray. Afterwards, the

specimens were fixed with 4% paraformaldehyde, decalcified with 10% ETDA, and sliced into 5 μm thick sections for next experiments.

1.16 Histological analysis

To evaluate the morphology of articular surface and subchondral bone and proteoglycan loss, the slices were stained by safranin/F fast green staining and hematoxylin and eosin (HE) staining in accordance with the manufacturer's protocol. Then, the slices were imaged via an automatic digital slide scanning system, which were finally evaluated by several well-accepted osteoarthritis and synovitis scoring system.

1.17 Immunohistochemistry

Sections were incubated in 0.1% trypsin for 20 min, followed by blocking with goat serum albumin (10%) for 1 h. After co-incubation with primary antibodies against Aggrecan, MMP13, collagen II, and P21 overnight at 4 °C, the slices were incubated with the HRP-conjugated secondary antibody for 2 h. The slices were imaged, and quantitatively by positive cell rate.

1.18 Statistical analysis

Data were presented as mean ± standard deviation (SD) for $n \geq 3$ independent experiments. Statistical significance was calculated via one-way ANOVA with Tukey's test.

2. Supporting figures

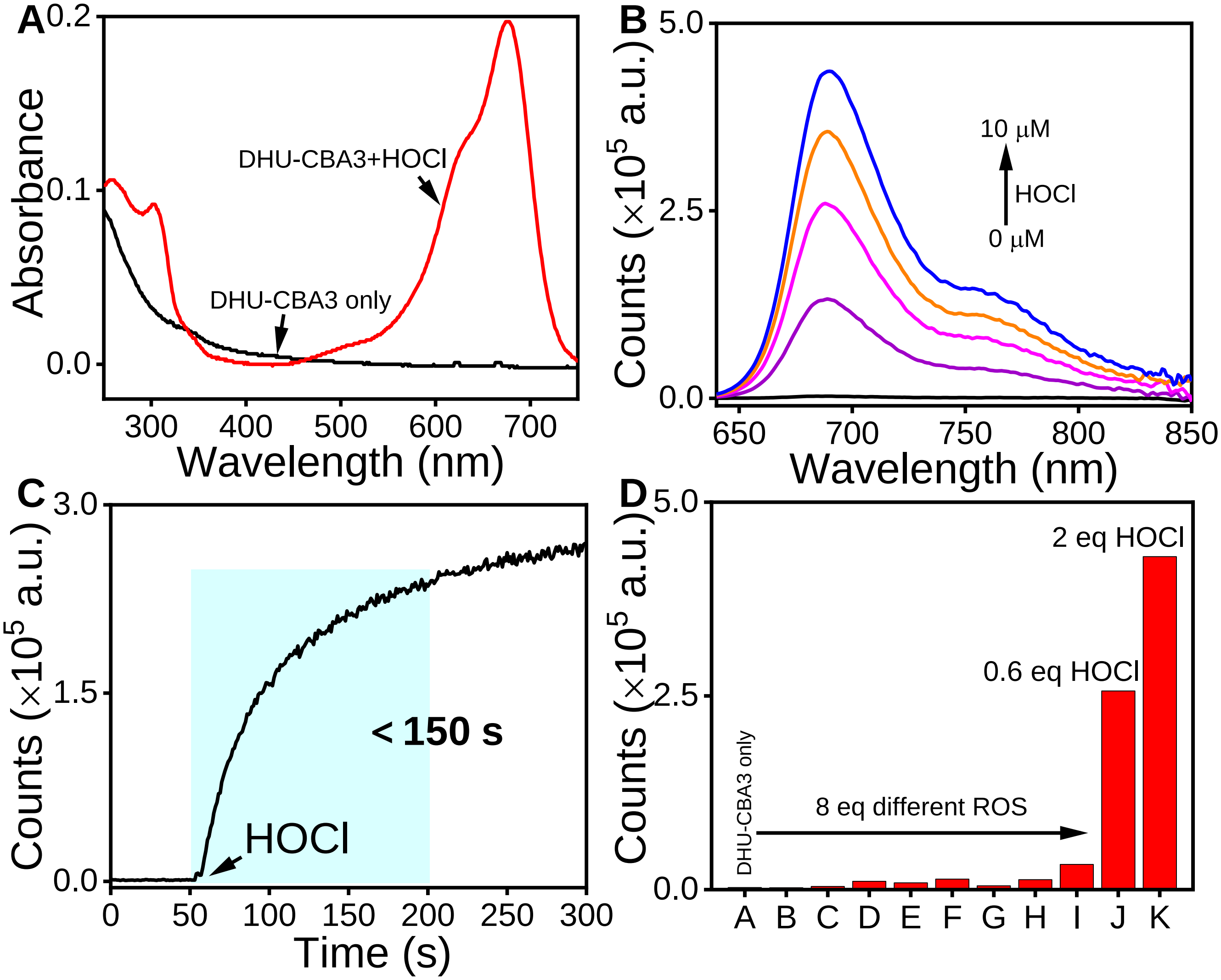

Figure S1. (A) Absorption spectra of DHU-CBA3 (5 μM) before and after addition of HOCl (10 μM). (B) Fluorescent spectra of DHU-CBA3 (5 μM) before and after addition of different concentrations of HOCl (0, 1, 3, 5, 10 μM). (C) The time-dependent changes in fluorescent intensity of DHU-CBA3 (5 μM) at 686 nm after adding HOCl (10 μM). (D) Fluorescent intensity of DHU-CBA3 (5 μM) at 686 nm after adding different ROS (40 μM) (from A to K): DHU-CBA3 only, H_2O_2, TBHP, $ROO^{\bullet}$, NO, $^{\bullet}OH$, $ONOO^{-}$, t-$BuOO^{\bullet}$, O_2^{-}, 3 and 20 μM HOCl. Time range 0 - 300 s; The data was recorded in PBS (10 mM, pH = 7.4, 1‰ N, N-dimethylformamide (DMF)); λ_{ex} = 620 nm.

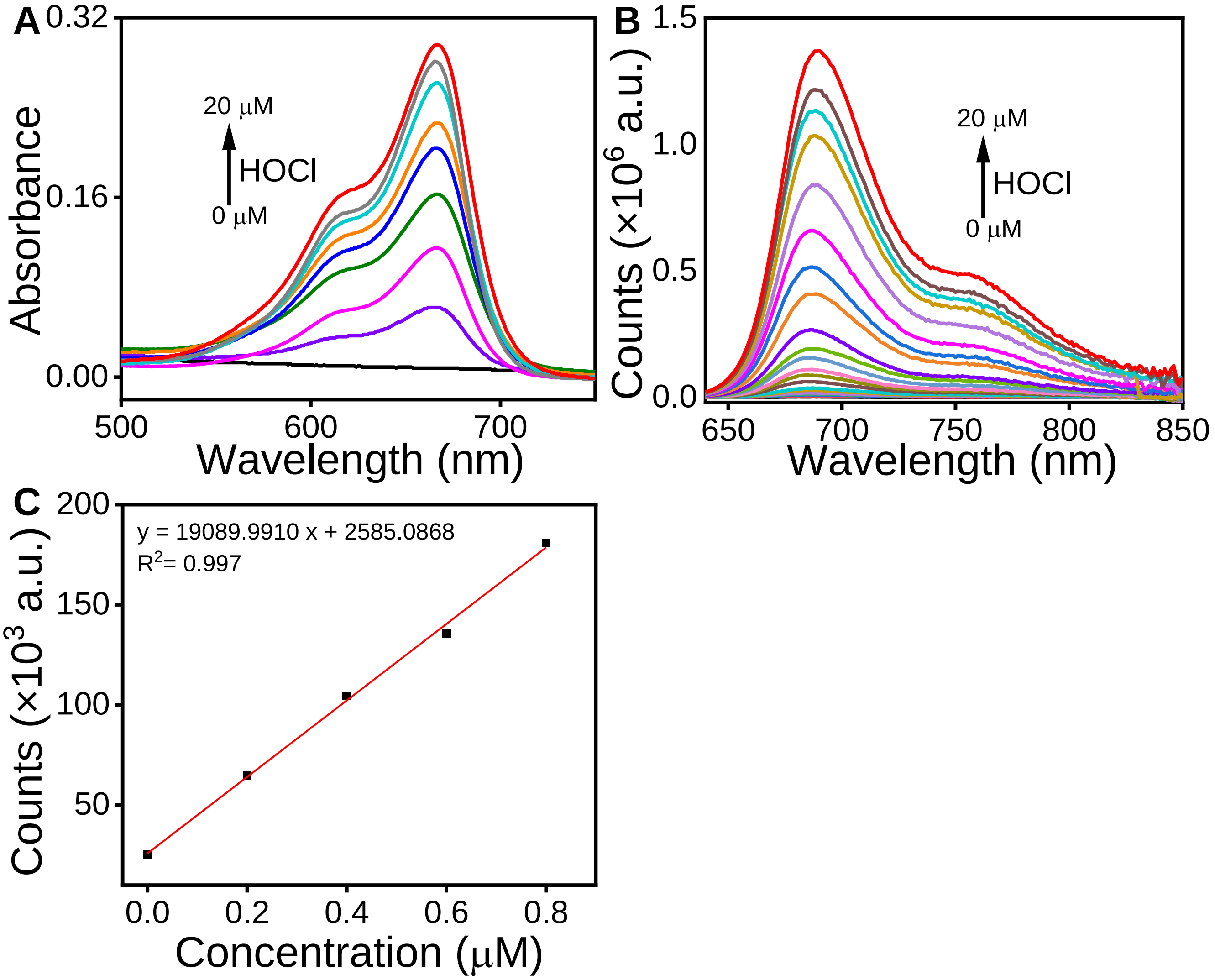

Figure S2. (A) The absorption spectra and (B) fluorescent spectra of DHU-CBA1 (5 μM) after addition different concentrations of HOCl (0 - 20 μM). (C) The linear relationship between the fluorescence intensity of DHU-CBA1 (5 μM) at 686 nm and the concentrations of HOCl (0.2 - 0.8 μM). The data was recorded in PBS. (10 mM, pH = 7.4, 1‰ DMF).

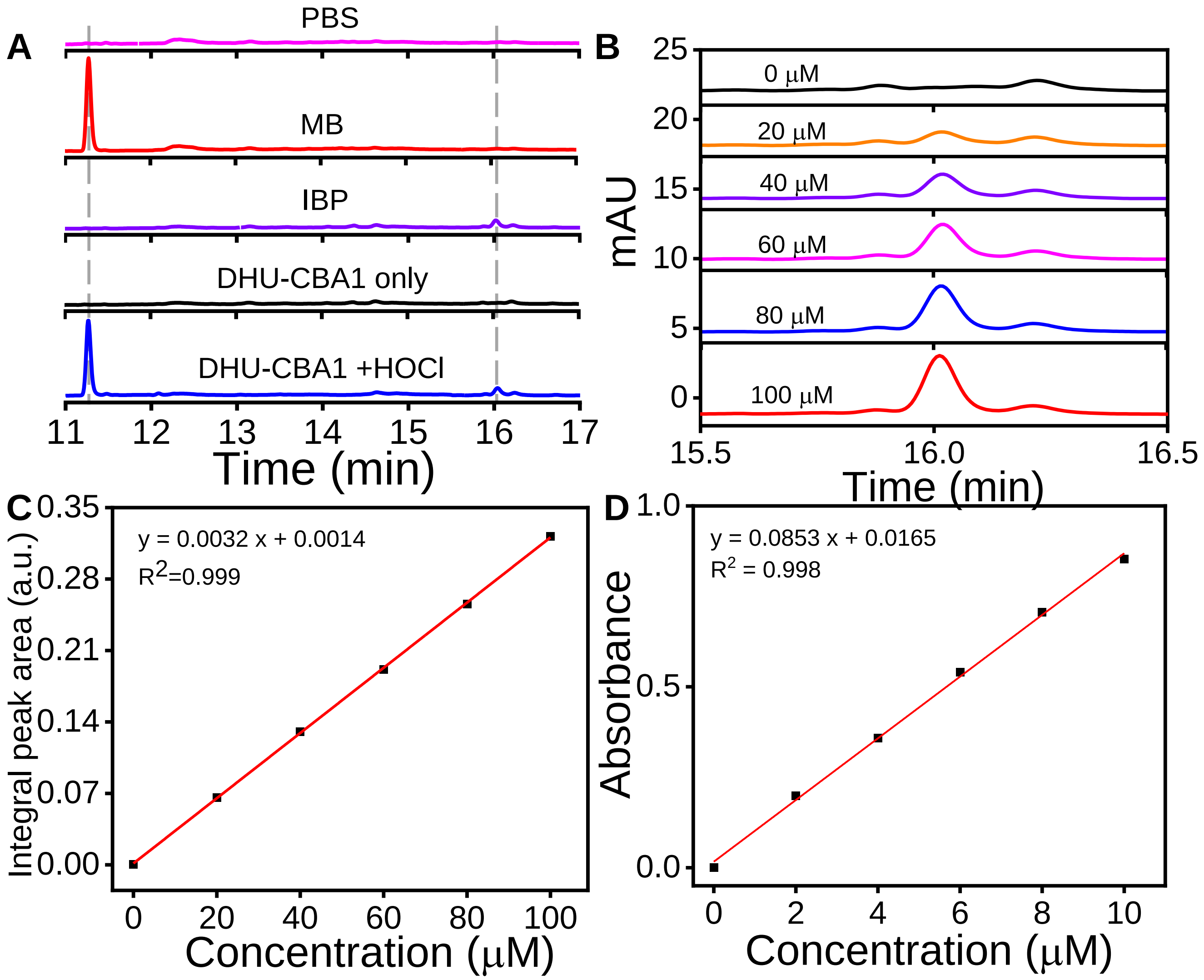

Figure S3. (A) HPLC analysis of the aqueous solution: PBS, 40 μM MB, 40 μM IBP, 40 μM DHU-CBA1only, 40 μM DHU-CBA1+200 μM HOCl. (B) HPLC analysis of the integral peak area of free IBP from 20 - 100 μM. (C) HPLC analysis of the linear fit of integral peak area of free IBP from 20 - 100 μM. (254 nm). (D) The linear relationship between the absorbance of DHU-CBA1 (5 μM) at 664 nm and the concentrations of HOCl (2 - 10 μM).

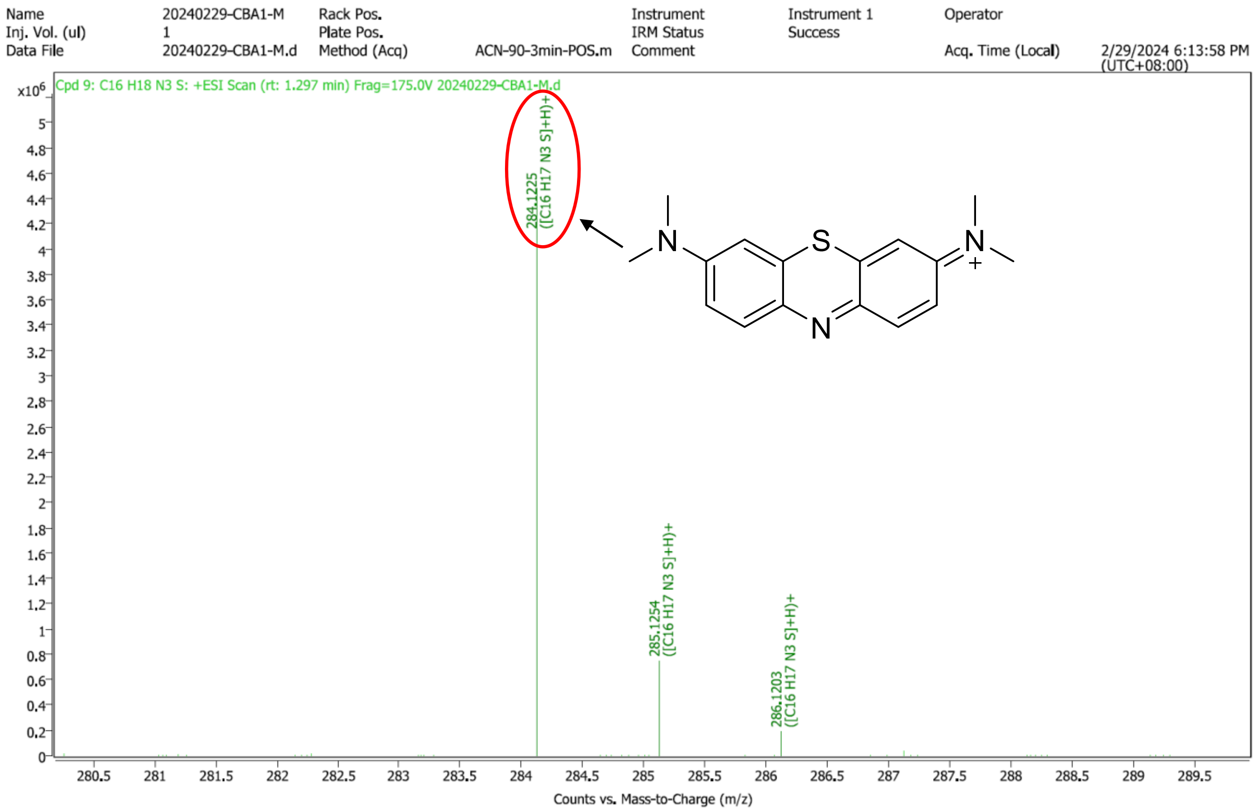

Figure S4. HRMS analysis of DHU-CBA1 (5 μM) after reacted with 20 μM HOCl.

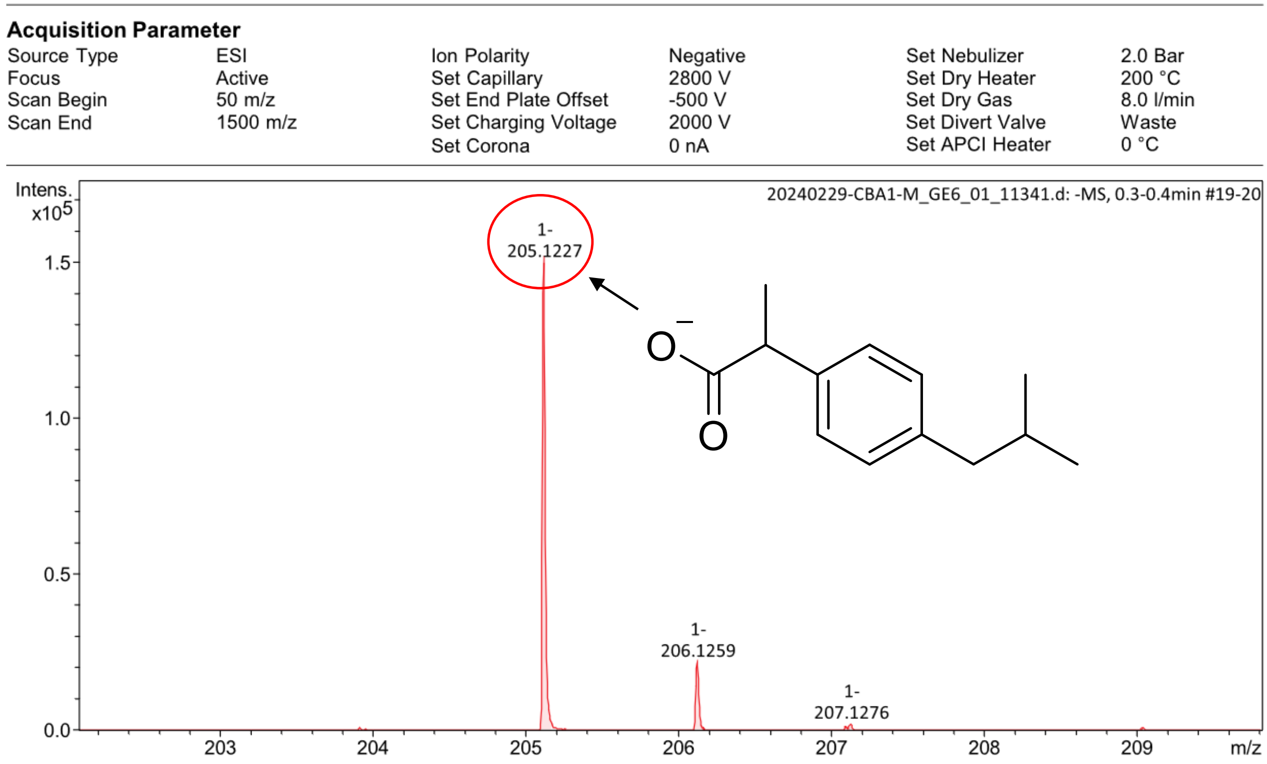

Figure S5. HRMS analysis of probe DHU-CBA1 (5 μM) after reacted with 20 μM HOCl.

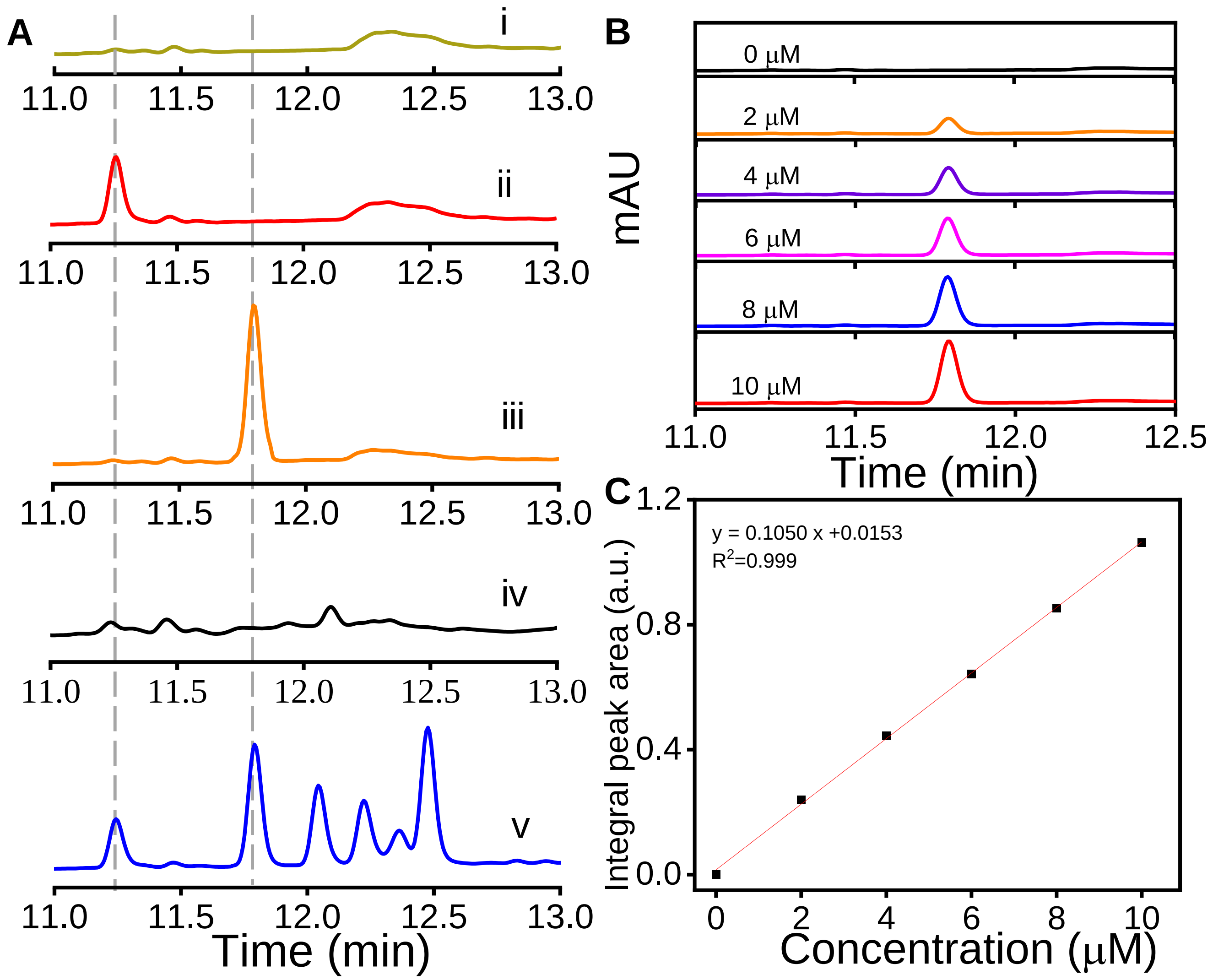

Figure S6. (A) HPLC analysis of the aqueous solution from (i) PBS, (ii) 5 μM MB, (iii) 5 μM pTA, (iv) 5 μM DHU-CBA3, (v) 5 μM DHU-CBA3+20 μM HOCl. (B) HPLC analysis of the integral peak area of free pTA from 2 - 10 μM. (C) HPLC analysis of the linear fit of integral peak area of free pTA from 2 - 10 μM (254 nm).

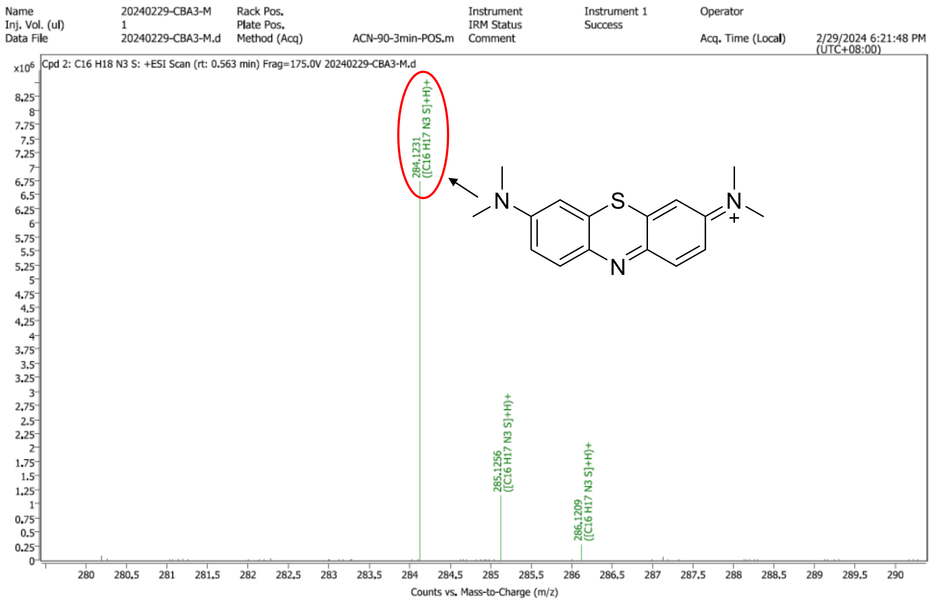

Figure S7. HRMS analysis of probe DHU-CBA3 (5 μM) after reacted with 10 μM HOCl.

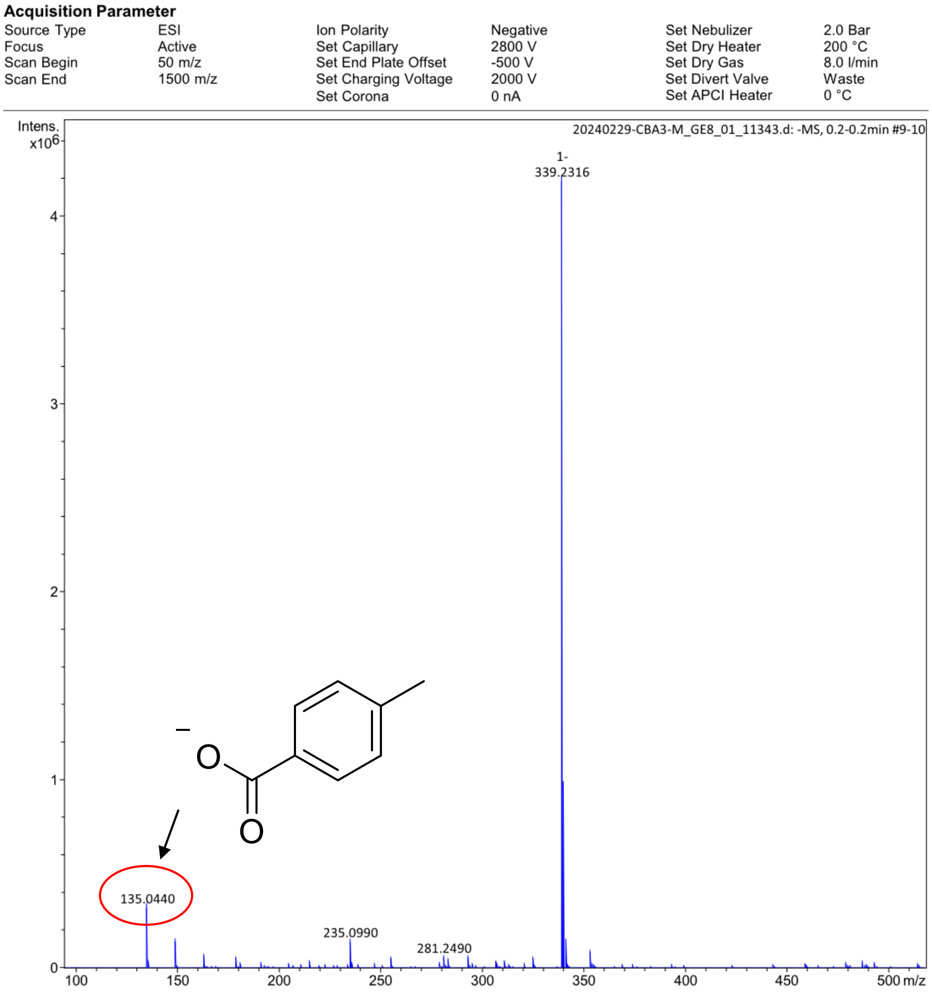

Figure S8. HRMS analysis of probe DHU-CBA3 (5 μM) after reacted with 10 μM HOCl.

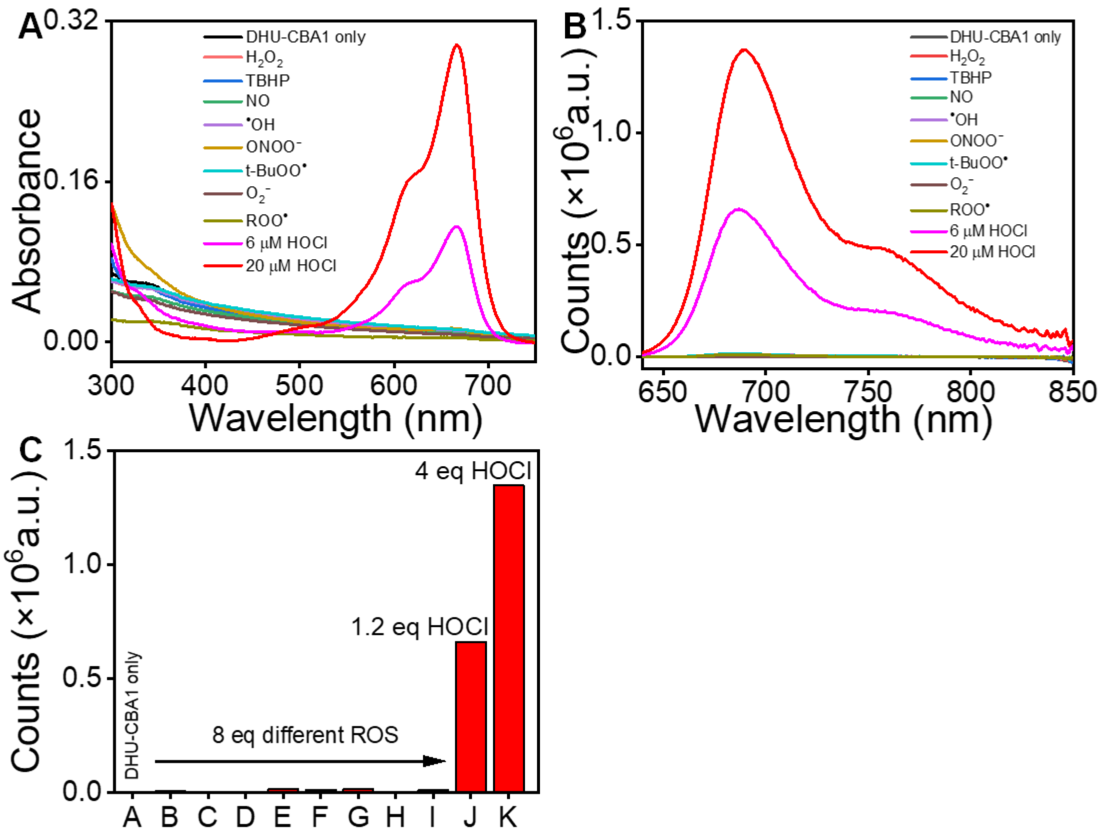

Figure S9. (A) The absorption spectra and (B) fluorescent spectra of DHU-CBA1 (5 μM) changes after adding different ROS (40 μM). (C) The fluorescent intensity of DHU-CBA1 (5 μM) at 686 nm after adding different ROS (40 μM) (from A to K): DHU-CBA1 only, H_2O_2, TBHP, ROO•, NO, •OH, $ONOO^-$, t-BuOO•, O_2^-, 6 and 20 μM HOCl. The data was recorded in PBS (10 mM, pH = 7.4, 1‰ DMF); λ_{ex} = 620 nm.

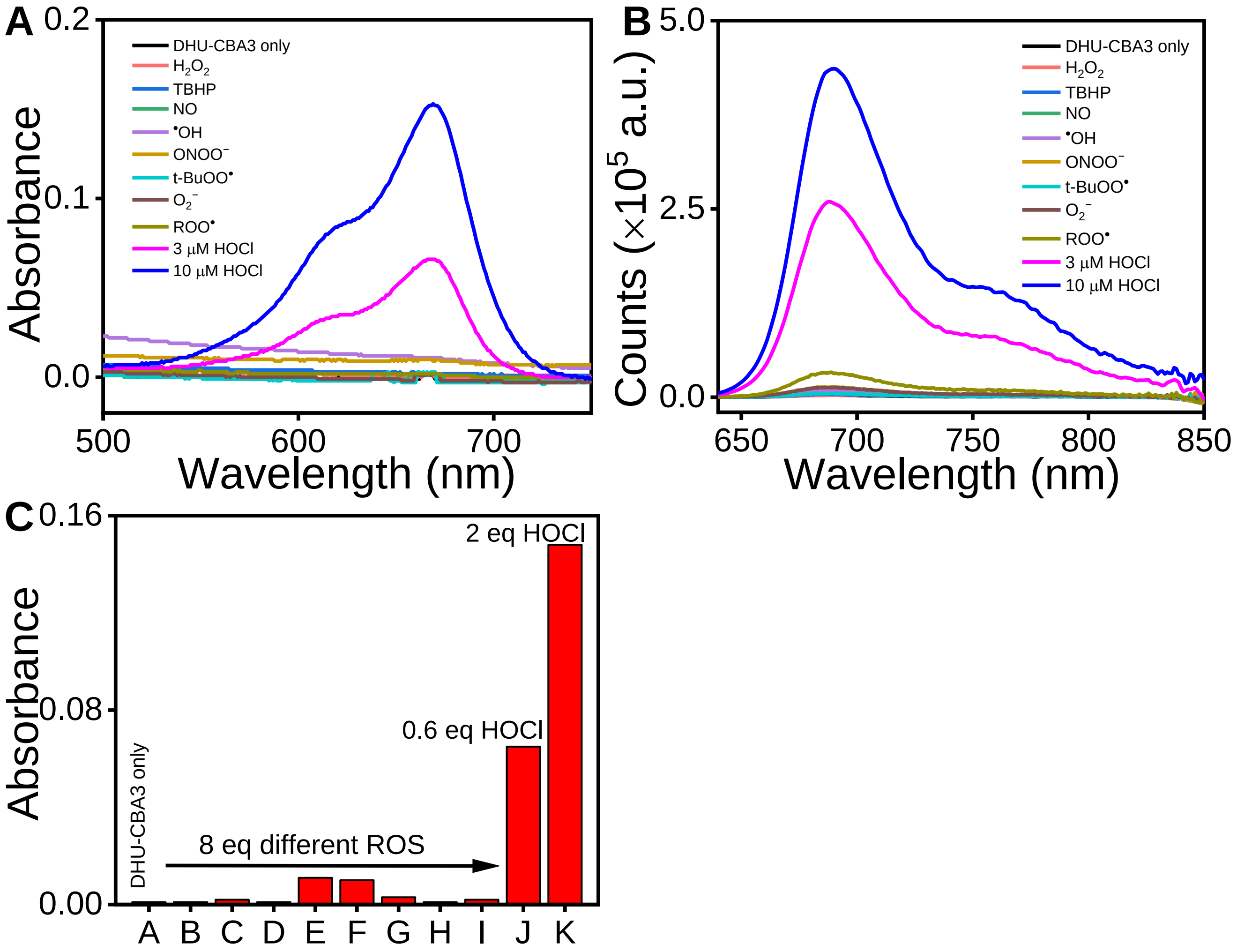

Figure S10. (A) The absorption spectra and (B) fluorescent spectra of DHU-CBA3 (5 μM) changes after adding different ROS (40 μM). (C) The absorbance of DHU-CBA3 (5 μM) at 664 nm after adding different ROS (40 μM) (from A to K): DHU-CBA3 only, H_2O_2, TBHP, $ROO^•$, NO, $^•OH$, $ONOO^-$, t-$BuOO^•$, O_2^-, 3 and 20 μM HOCl. The data was recorded in PBS (10 mM, pH = 7.4, 1‰ DMF); λ_{ex} = 620 nm.

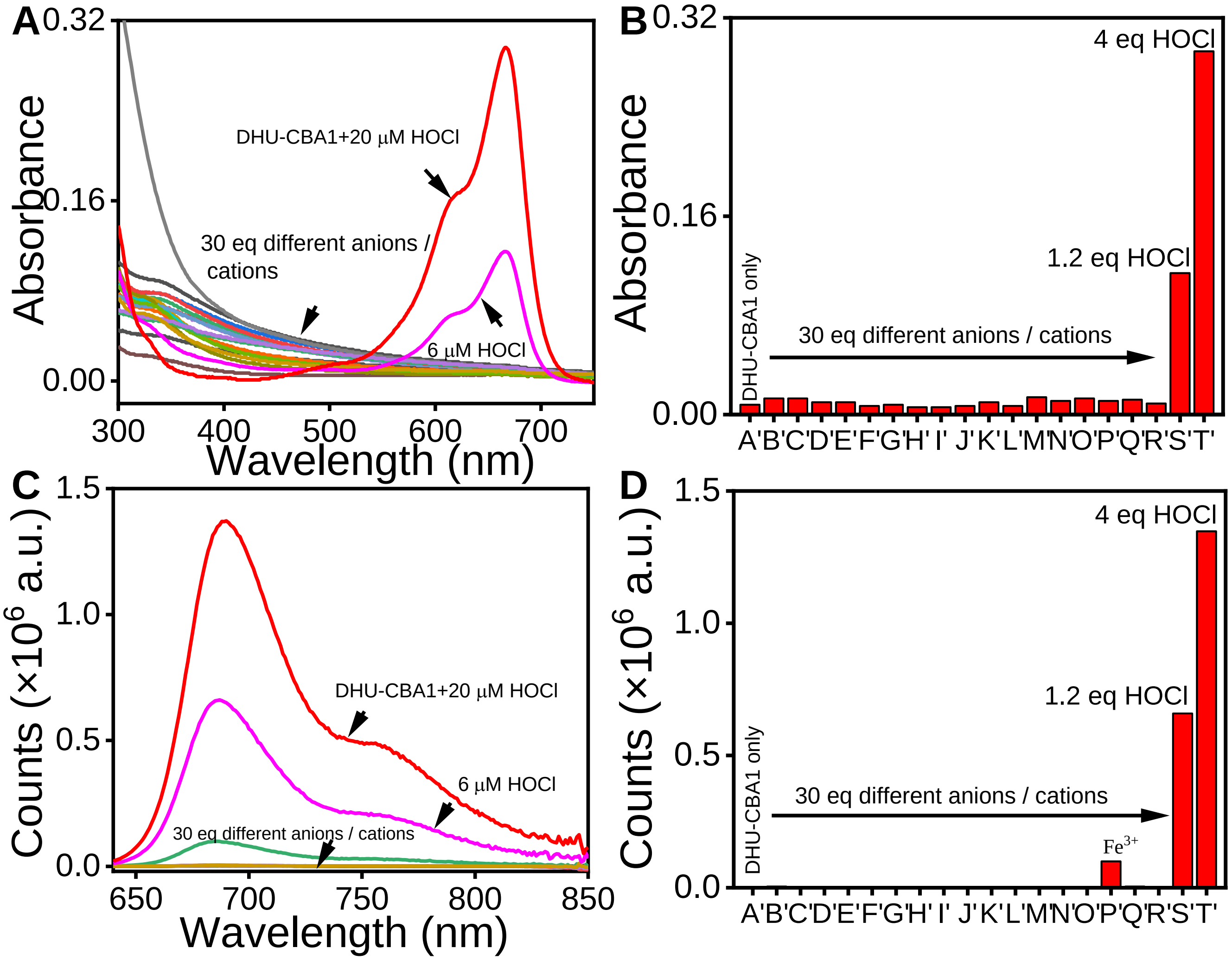

Figure S11. (A) The absorption spectra and (C) fluorescent spectra of DHU-CBA1 (5 μM) changes after adding various anions/cations (150 μM). (B) The absorbance DHU-CBA1 (5 μM) at 664 nm and (D) fluorescent intensity of DHU-CBA1 (5 μM) at 686 nm after adding various anions/cations (150 μM) (from (A') to (T')): DHU-CBA1 only, CH_3COO^-, CO_3^{2-}, SO_4^{2-}, F^-, Cl^-, I^-, NO_2^-, $S_2O_3^{2-}$, NH_4^+, Na^+, Mg^{2+}, Al^{3+}, K^+, Ca^{2+}, Fe^{3+}, Cu^{2+}, Ni^{2+}, 6 and 20 μM HOCl. The data was recorded in PBS (10 mM, pH = 7.4, 1‰ DMF); λ_{ex} = 620 nm.

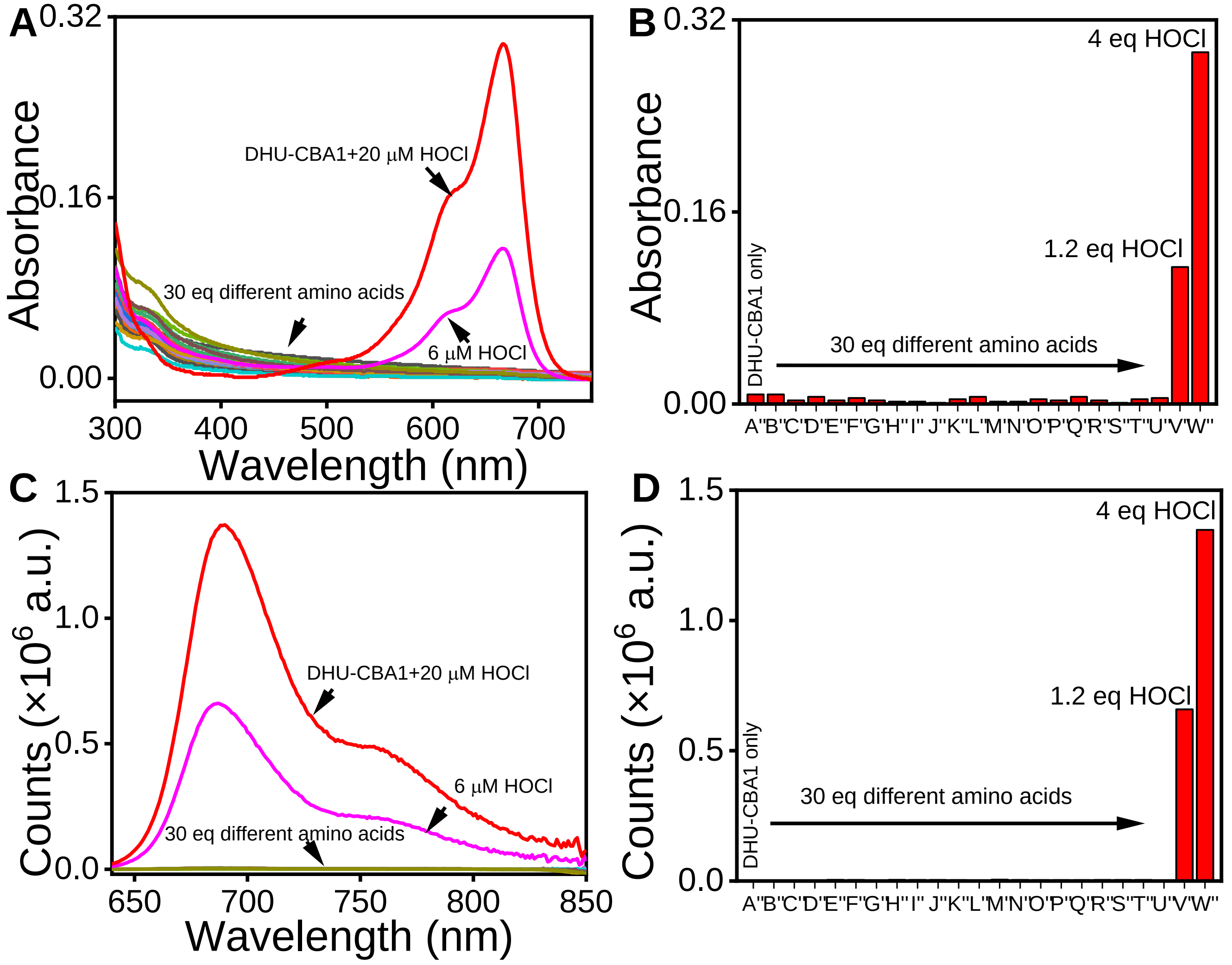

Figure S12. (A) The absorption spectra and (C) fluorescent spectra of DHU-CBA1 (5 μM) changes after adding various amino acids (150 μM). (B) The absorption strength of DHU-CBA1 (5 μM) at 664 nm and (D) fluorescent intensity of DHU-CBA1 (5 μM) at 686 nm after adding various amino acids (150 μM) (from (A'') to (T'')): DHU-CBA1 only, Leu, Pro, Gly, Gln, Glu, Met, Lys, Trp, Ser, Thr, Asp, Ile, Val, His, Ala, Cys, Phe, Asn, Tyr, Arg, 6 and 20 μM HOCl. The data was recorded in PBS (10 mM, pH = 7.4, 1‰ DMF); λ_{ex} = 620 nm.

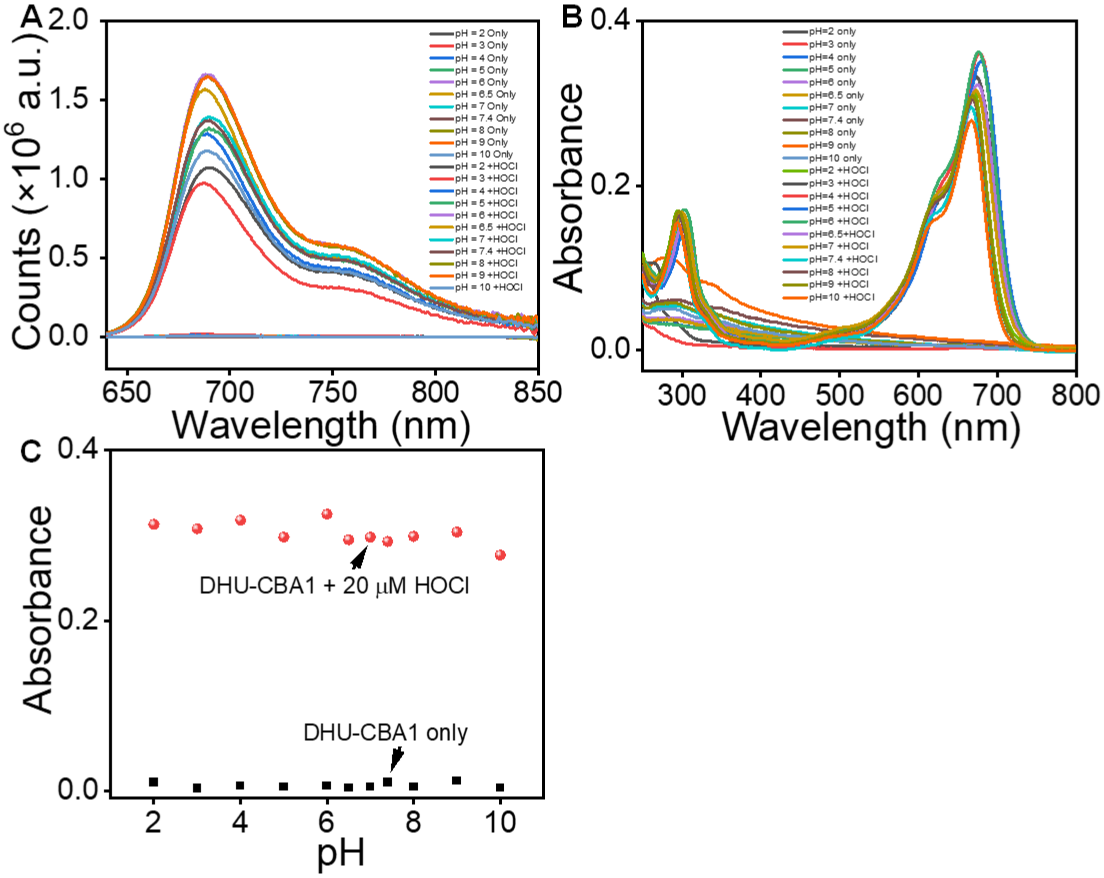

Figure S13. (A) The fluorescent spectra and (B) absorption spectra of DHU-CBA1 (5 μM) changes after adding 20 μM HOCl in buffer with different pH (2-10). (C) The absorption strength of DHU-CBA1 (5 μM) at 664 nm before and after adding 20 μM HOCl in buffer with different pH (2-10). The data was recorded in PBS (10 mM, pH = 7.4, 1‰ DMF); λ_{ex} = 620 nm.

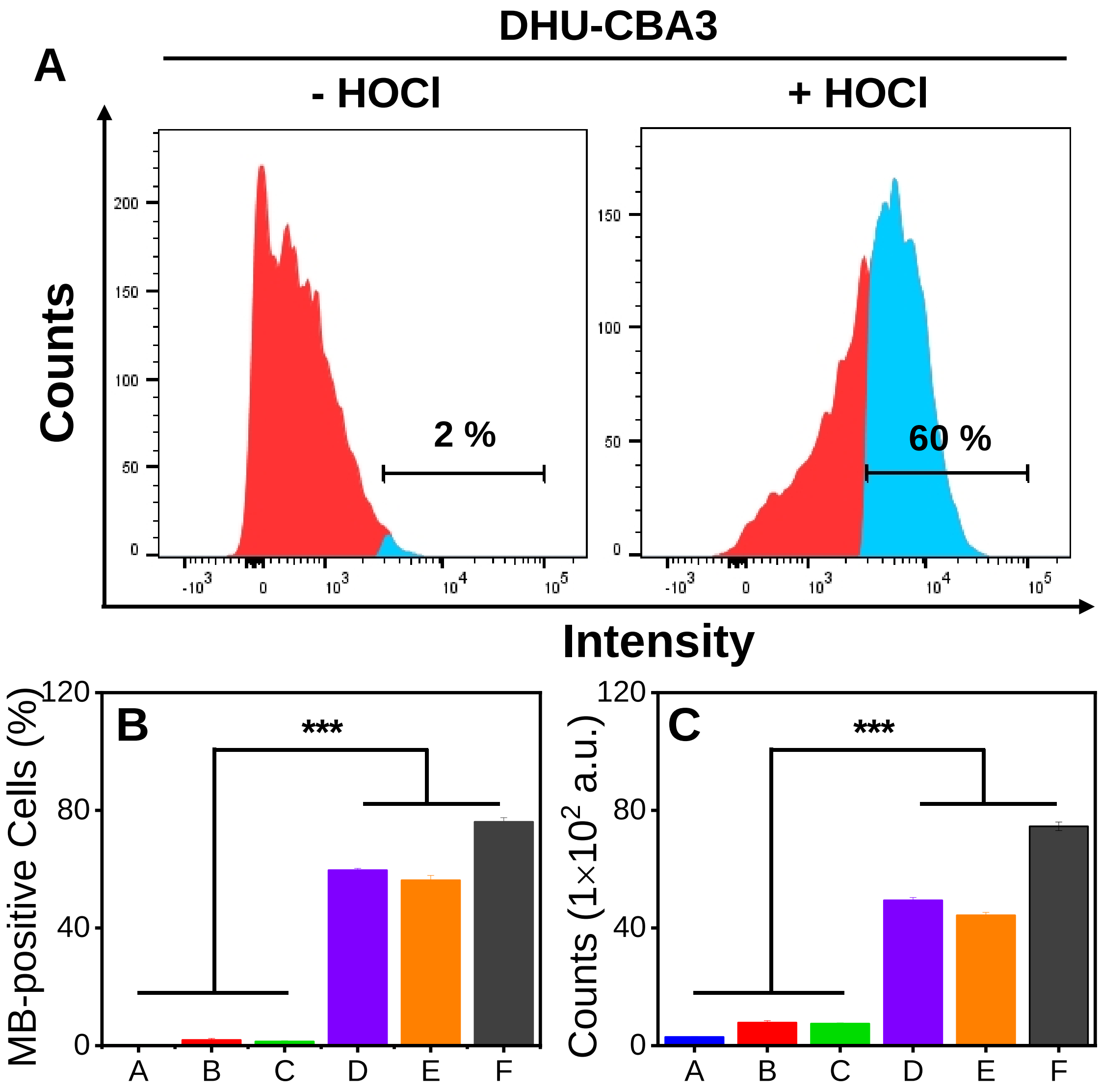

Figure S14. (A) Flow cytometry analysis of chondrocytes cells uptake and activation with 10 μM DHU-CBA3 and 10 μM DHU-CBA3 + HOCl, respectively. (B) The average fluorescence intensity output of the groups (A - F: CTRL, DHU-CBA3, DHU-CBA1, DHU-CBA3 + HOCl, DHU-CBA1 + HOCl and MB). (C) The relative fluorescence intensity output of the groups (A - F: CTRL, DHU-CBA3, DHU-CBA1, DHU-CBA3 + HOCl, DHU-CBA1 + HOCl and MB). Values are the mean ± SD. for n = 3, *** p < 0.001.

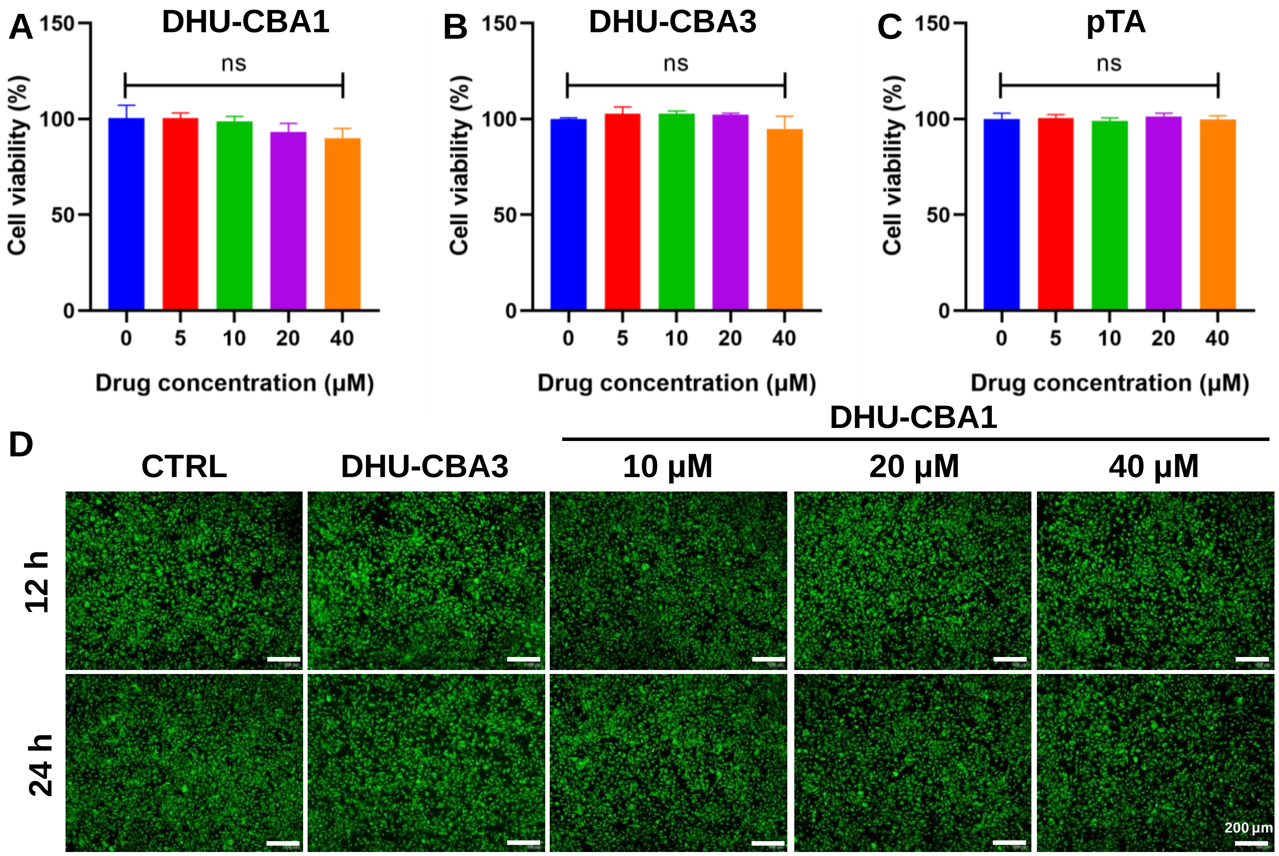

Figure S15. (A-C) The CCK-8 results of the cell viability in chondrocytes incubated with DHU-CBA1, DHU-CBA3, and pTA at different concentrations (5, 10, 20, 40 μM) for 24 h. (D) The dead/live staining reveals the chondrocyte toxicity of DHU-CBA1 (10, 20, 40 μM) and DHU-CBA3 (40 μM) for 12 h and 24 h. Data are presented as means ± SD.

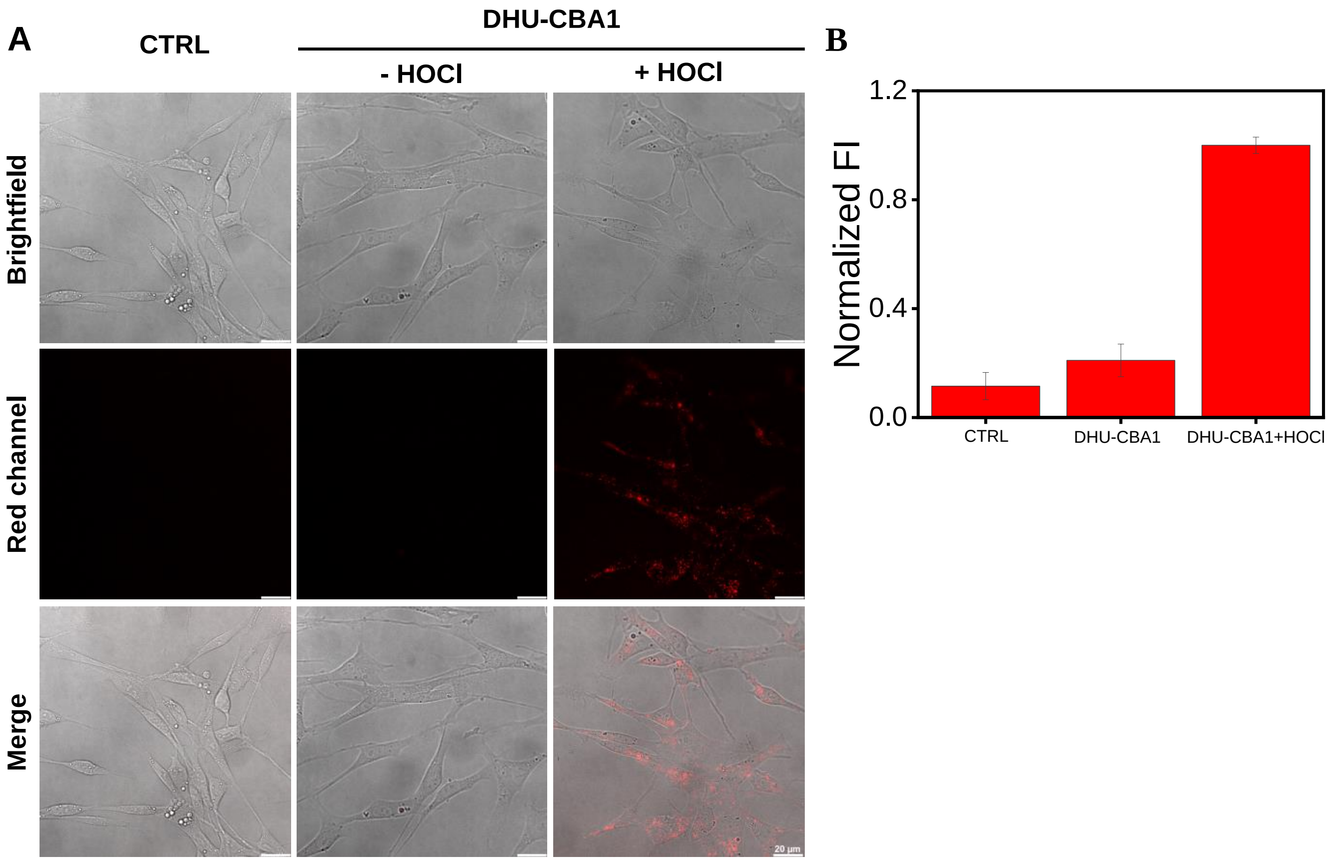

Figure S16. (A) CLSM images of chondrocytes cells incubated with medium (CTRL), 10 μM DHU-CBA1(1 h), and 10 μM DHU-CBA1 (1 h) + 40 μM HOCl (15 min subsequently) respectively. (B) The fluorescence intensity output of the groups (CTRL, 10 μM DHU-CBA1(1 h), and 10 μM DHU-CBA1 (1 h) + 40 μM HOCl (15 min subsequently)). (λ_{ex}= 633 nm, Red channel: 700 ± 50 nm, Scale bar = 20 μm).

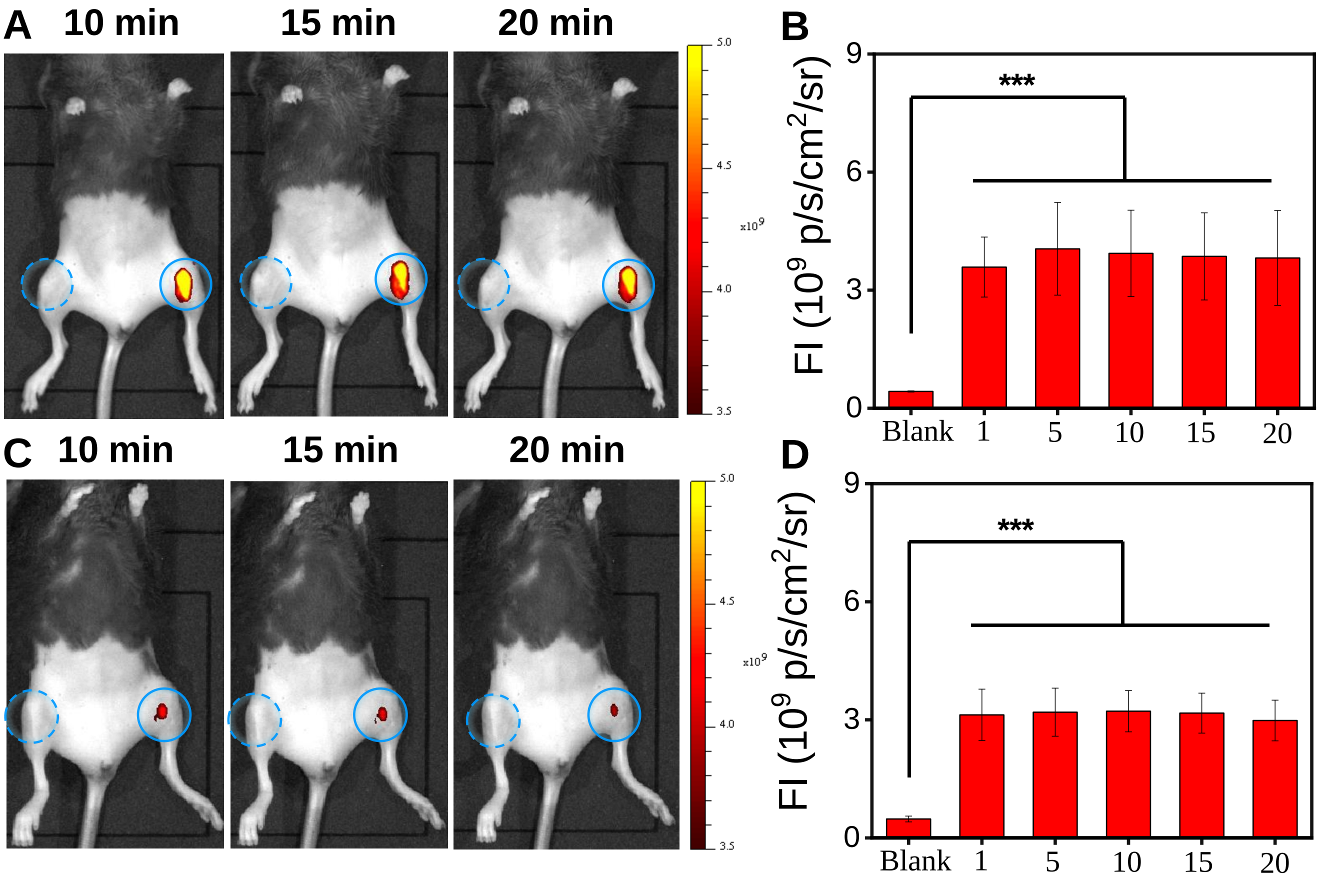

Figure S17. (A) The fluorescence images with (A) DHU-CBA1 and (C) DHU-CBA3 (10 μL × 0.5 mM) for different times (10, 15 and 20 min) of OA model in vivo. The average fluorescence intensity in the OA mice model injected intra-articular with (B) DHU-CBA1 and (D) DHU-CBA3 (10 μL × 0.5 mM) at different times (0, 1, 5, 10, 15 and 20 min). Values are the mean ± SD. for n = 3, *** $p < 0.001$.

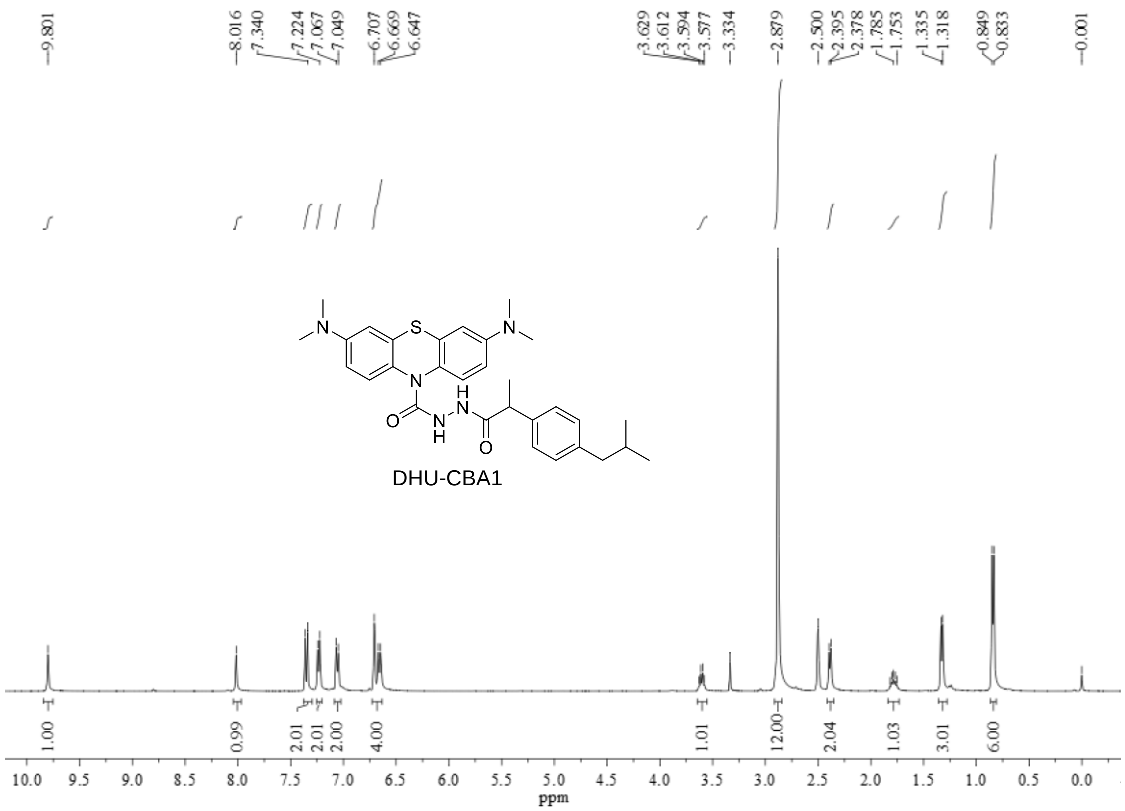

Figure S18. ^{1}H NMR of **DHU-CBA1** in DMSO-d_6.

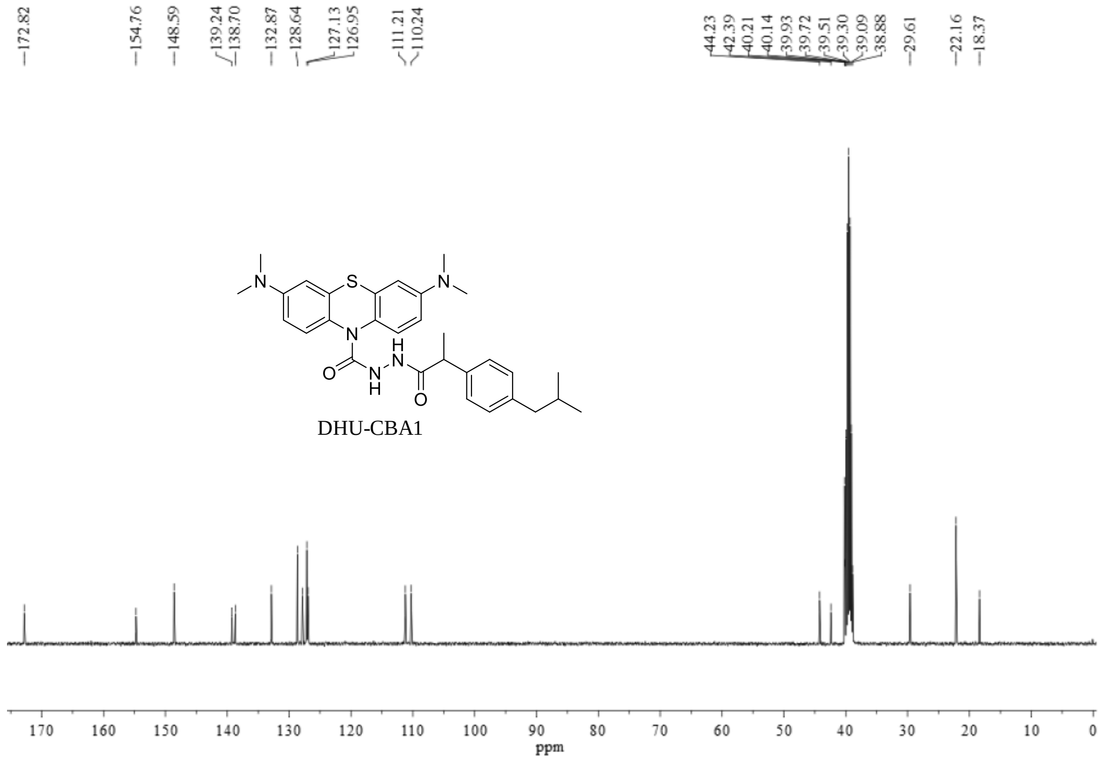

Figure S19. ^{13}C NMR of **DHU-CBA1** in DMSO-d_6.

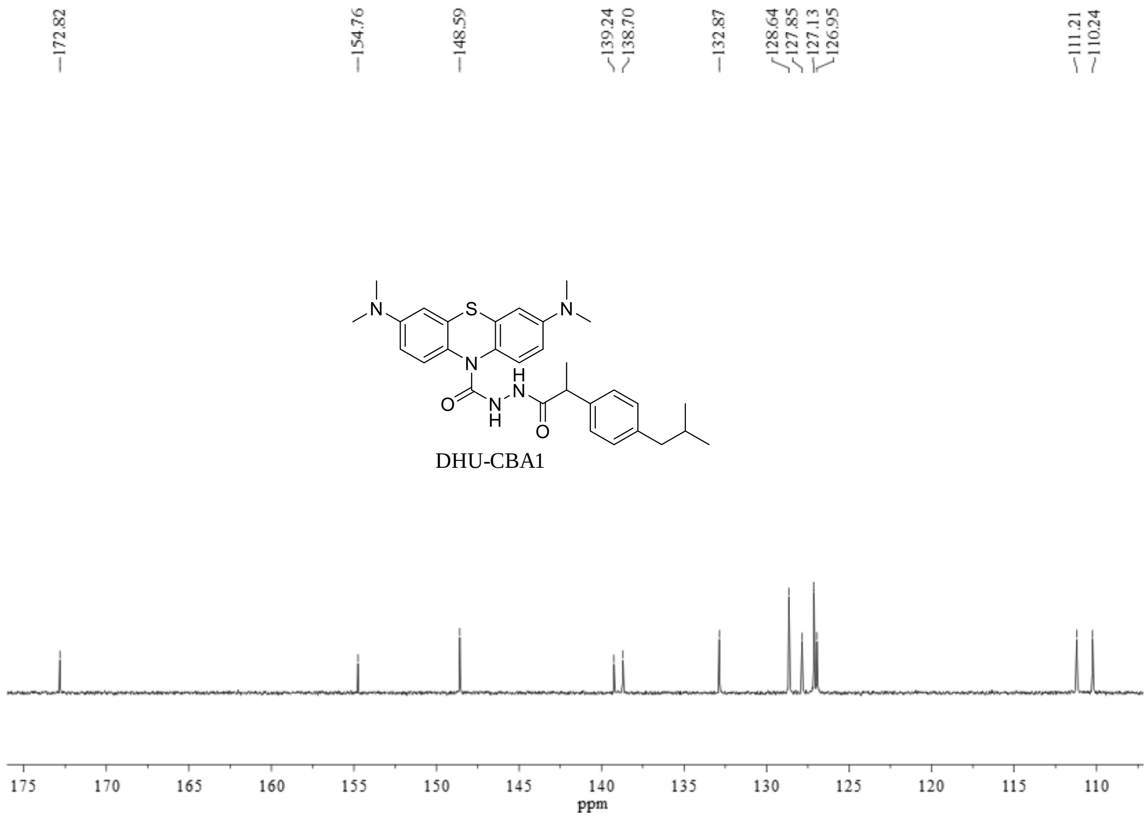

Figure S20. ^{13}C NMR of **DHU-CBA1** in DMSO-d_6.

Acquisition Parameter

Source Type	ESI	Ion Polarity	Positive	Set Nebulizer	2.0 Bar
Focus	Active	Set Capillary	4500 V	Set Dry Heater	200 °C
Scan Begin	50 m/z	Set End Plate Offset	-500 V	Set Dry Gas	8.0 l/min
Scan End	3000 m/z	Set Charging Voltage	2000 V	Set Divert Valve	Waste
		Set Corona	0 nA	Set APCI Heater	0 °C

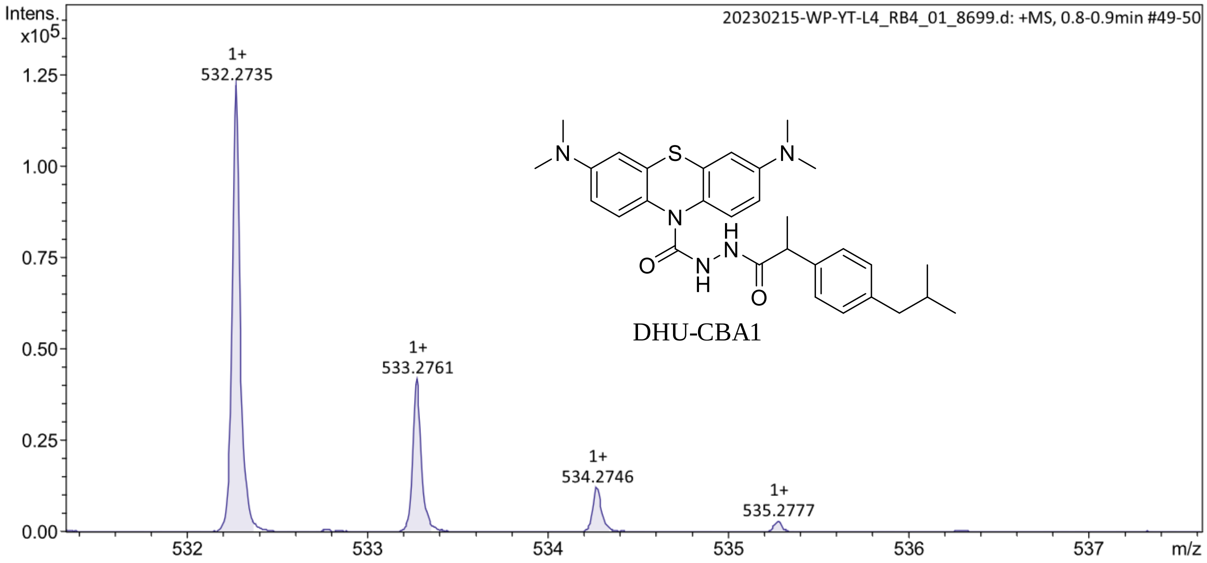

Figure S21. HRMS of **DHU-CBA1**.

3. References

1. Wei P, Yuan W, Xue F, Zhou W, Li R, Zhang D, et al. Deformylation reaction-based probe for in vivo imaging of HOCl. Chem Sci. 2018; 9: 495-501.
2. Chen Y, Long Z, Wang C, Zhu J, Wang S, Liu Y, et al. A lysosome-targeted near-infrared fluorescent probe for cell imaging of Cu^{2+}. Dyes Pigm. 2022; 204: 110472.
3. Wang B, Lu Z-N, Song M-X, He X-W, Hu Z-C, Liang H-F, et al. Single-component dual-functional autoboost strategy by dual photodynamic and cyclooxygenase-2 inhibition for lung cancer and spinal metastasis. Adv Sci. 2024; 11, 12: 2303981.
4. Wei P, Liu L, Wen Y, Zhao G, Xue F, Yuan W, et al. Release of amino- or carboxy-containing compounds triggered by HOCl: Application for imaging and drug design. Angew Chem Int Ed. 2019; 58: 4547-51.
5. Liu L, Liu F, Liu D, Yuan W, Zhang M, Wei P, et al. A smart theranostic prodrug system activated by reactive oxygen species for regional chemotherapy of metastatic cancer. Angew Chem Int Ed. 2022; 61: e202116807.
